# Buccal and sublingual lyophilised naloxone for opioid overdose reversal: A Phase 1 open-label, random-sequence crossover pharmacokinetic study protocol in healthy volunteers (NalBuc)

**DOI:** 10.1371/journal.pone.0347370

**Published:** 2026-10-27

**Authors:** Mariana Gonzalez Utrilla, Alexander Baker, Edward Chesney, Kim Lee, Richard Emsley, John Strang

**Affiliations:** 1 National Addiction Centre, Institute of Psychiatry, Psychology and Neuroscience (IoPPN), King’s College London, London, United Kingdom; 2 South London and Maudsley NHS Foundation Trust, London, United Kingdom; 3 Department of Biostatistics and Health Informatics, Institute of Psychiatry, Psychology and Neuroscience, King’s College London, London, United Kingdom; 4 King’s Clinical Trials Unit, King’s College London, London, United Kingdom; University of Toronto, CANADA

## Abstract

**Background:**

Opioid overdose remains a major global public health emergency, with naloxone being an essential response. Benefit from existing naloxone formulations is limited by barriers to acceptability, fixed dosing, and poor real-world carriage. This study describes the protocol for a Phase 1 exploratory investigation of a novel ultra-portable lyophilised naloxone wafer designed to facilitate constant carriage and enable rapid mucosal absorption.

**Methods:**

This open-label, randomised, random-sequence, five-way crossover pharmacokinetic (PK) exploratory study will recruit 12–15 healthy adults at a UK clinical research facility. Participants will receive five treatments, each separated by ≥36 hours: buccal naloxone wafer 2 mg, buccal wafer 4 mg, sublingual wafer 2 mg, intramuscular naloxone 0.4 mg, and intranasal naloxone 1.8 mg (Nyxoid®). Blood samples will be collected up to 240 minutes post-dose for PK analysis via validated LC-MS/MS methods. The primary endpoint is time to maximum plasma concentration (Tmax). Secondary endpoints include Maximum Plasma Concentration (Cmax), Time to 50% of maximum concentration (T50%), Area Under the Curve from time zero to 15 minutes (AUC_0–15_), Area Under the Curve from time zero to infinity (AUCinf), and safety/tolerability. Dose-proportionality and buccal–sublingual comparisons will be explored. Trial Registration:REC: 25/LO/0701; MHRA CTA (IRAS): 1011060. ISRCTN67173532.

**Results & Significance:**

This study will characterise the absorption profile, early exposure, and relative bioavailability of a novel naloxone wafer formulated for rapid buccal and sublingual administration. Findings will inform whether this technology could provide a practical, effective alternative to current injectable and intranasal products, with potential to substantially improve naloxone carriage and timely overdose response, and will guide the design of a subsequent, larger confirmatory study.

## Introduction

Opioid overdoses continue to represent a critical global public health challenge, accounting for an estimated 150,000 deaths annually worldwide and approximately 3,000 deaths across the United Kingdom [1,2]. This crisis has intensified over the past two decades, driven by multiple factors including the proliferation of highly potent synthetic opioids, poly-substance use patterns, and periodic fluctuations in the purity and composition of illicit drug supplies [3–5].

These deaths are largely preventable through the timely administration of naloxone, which has been used clinically since the 1970s with a well-established safety profile. Originally approved for intravenous, intramuscular, and subcutaneous administration, it has been included in the World Health Organisation’s Model List of Essential Medicines since 1983 [6]. The mechanism of action involves competitive antagonism at mu-opioid receptors, rapidly displacing opioid agonists and reversing their effects, particularly respiratory depression, within 2–3 minutes of administration [7,8].

Since most opioid overdoses occur in community settings rather than in clinical settings, take-home naloxone (THN) programmes were developed to place naloxone directly in the hands of people who use opioids, their peers, and other likely bystanders [9–13]. THN has been widely implemented in the UK and internationally, and evidence consistently shows that it increases timely bystander intervention and reduces fatal overdose by enabling rapid administration before emergency services arrive [9,10,12].

### Limitations of current naloxone formulations

Current naloxone formulations face significant limitations that constrain their effectiveness in real-world emergencies [14,15]. Injectable formulations, while highly effective, have low acceptability/take-up by lay people as they require carrying syringes and ampoules, raise concerns about needlestick injuries, and more extensive training [16,17].

More recently, intranasal formulations (Nyxoid®, Narcan®, Ventizolve ™) have been introduced to facilitate lay administration by eliminating the need for needles and injection technique, allowing bystanders to deliver naloxone through a simple nasal spray device that requires minimal training [18,19]. These formulations addressed key barriers associated with injectable naloxone and have substantially broadened the potential pool of bystanders who can confidently deliver naloxone during an overdose emergency [9,20–23].

Despite representing an important step forward, several factors limit their universal applicability. First, nasal absorption can be affected by upper respiratory infections, nasal congestion, or structural damage to nasal passages [24,25]. Secondly, current intranasal products deliver a fixed, all-or-nothing dose that cannot easily be titrated to individual needs or overdose severity [26,27]. If the predetermined dose proves insufficient for complete reversal, administering additional doses requires separate devices, creating delays and logistical challenges [27].

Across all current formulations, the fundamental challenge remains that even when naloxone is provided to at-risk individuals, carriage rates remain unacceptably low.

### The critical problem of poor carriage rates

Even though THN programs have demonstrated effectiveness in reducing overdose mortality [10,20], their life-saving potential is critically dependent on the medication being immediately accessible when an overdose occurs [28].

A critical determinant of survival is not merely the willingness of a witness to administer naloxone, but also its actual immediate availability and prompt administration as soon as the life-threatening overdose is identified, typically in community settings [29]. Current evidence indicates that only 10–20% of individuals provided with naloxone consistently carry it on their person [30,31]. This low carriage rate substantially reduces the likelihood of naloxone being available during an overdose emergency.

### The dose dilemma and the need for flexible dosing

An emerging challenge in overdose response is determining optimal naloxone dosing in an era of increasingly potent synthetic opioids [32]. Underdosing risks inadequate reversal and continued respiratory depression, while overdosing may precipitate severe acute opioid withdrawal in individuals who are opioid-dependent [27], potentially causing significant distress and reducing future willingness to accept overdose interventions [19].

Current fixed-dose formulations force an “all-or-nothing” approach that cannot be easily adapted to individual circumstances [26]. The ideal naloxone formulation would allow flexible, titratable dosing that can be adjusted to achieve adequate reversal while minimising precipitated withdrawal [27]. A formulation that can deliver naloxone in modular units, where multiple doses can be administered sequentially if needed, offers significant advantages.

### Critical pharmacokinetic (PK) considerations for emergency naloxone

The PK profile of naloxone formulations for emergency overdose reversal must meet specific criteria that differ from typical medication development priorities [33–35].

First, rapid absorption is essential. During opioid overdose, respiratory depression progresses quickly, and delays of even a few minutes in achieving therapeutic naloxone concentrations can result in fatal hypoxia or permanent neurological damage [36,37]. The time to reach maximum plasma concentration (T_max_) and, arguably more importantly, the time to reach clinically relevant concentrations (which may occur well before Maximum Plasma Concentration C_max_) can determine how quickly an overdose victim begins to breathe adequately again [33,34,38]. We have previously included examination of Time to 50% of maximum concentration (T_50%_) to enable this added consideration [35]. Formulations with slower absorption, even if ultimately achieving high bioavailability, may be less suitable for emergency use. Secondly, sufficient peak concentrations (C_max_) are needed to ensure adequate receptor occupancy for opioid reversal [32,33,35,38,39].

### Buccal and sublingual delivery as a promising alternative

Buccal and sublingual drug delivery represents a promising alternative route that has been successfully implemented for other emergency medications, such as midazolam for seizures [40–42]. The buccal mucosa offers several advantages: it avoids first-pass hepatic metabolism, provides direct systemic absorption through the rich vasculature draining into the internal jugular vein, and enables simple administration that can be performed by lay persons [42–44]. Previous research in animal models has demonstrated 70% bioavailability of naloxone via buccal administration compared to 0.3% via the oral route, with maximum plasma levels reached within 15 minutes [45]. Compared to the buccal mucosa, the sublingual mucosa has a thinner epithelium (approximately 100–200 μm versus 500–800 μm for buccal mucosa) and higher permeability, which may enable faster drug absorption and shorter time to peak plasma concentration [46].

Our research group has developed a novel ultra-portable, rapid-dispersal naloxone formulation in buccal wafer form using established lyophilisation technology. Preliminary feasibility studies have demonstrated that these wafers can be manufactured to disintegrate within seconds, and also that they appear stable over time whilst stored [41]. Our novel product is packaged in a credit-card format suitable for constant carriage in a wallet or purse.

The selection of 2 mg and 4 mg for the novel buccal formulation was informed by two key considerations. First, preclinical data in animal models have demonstrated approximately 70% bioavailability of naloxone via the buccal route, with peak plasma levels achieved within approximately 15 minutes [45]. Given that the approved IM comparator delivers 0.4 mg and the intranasal comparator (Nyxoid®) delivers 1.8 mg, doses of 2 mg and 4 mg were selected to bracket and exceed the intranasal reference dose while accounting for the expected, but as yet uncharacterised, buccal bioavailability in humans. Second, both doses were reviewed and approved by the MHRA as part of the Clinical Trial Authorisation (CTA) process (IRAS: 1011060), including a satisfactory assessment of the supporting toxicology dossier.

The PK profile of this novel formulation has not yet been characterised in humans. Understanding the absorption rate, bioavailability, and dose-proportionality of buccal naloxone wafers is essential before progressing to real-world implementation.

The primary objective of this exploratory study is to characterise and compare the PK profile of this buccal naloxone formulation in healthy volunteers with approved formulations (intramuscular naloxone ampoule as the primary reference and Nyxoid® intranasal spray as the secondary reference).

The secondary objectives are (1) to collect initial data on the dose-proportionality of the novel formulation by comparing plasma exposure of two different buccal doses (2 mg and 4 mg); and (2) to assess the safety and tolerability of the novel buccal formulation.

## Materials and methods

### Study design

This is a Phase 1 Clinical Trial of Investigational Medicinal Product (CTIMP) PK study conducted in the United Kingdom. The study employs an open-label, random-sequence, five-way cross-over design in which each participant receives all five treatments in a sequence determined by randomisation schedules generated by King’s Clinical Trials Unit:

- Buccal naloxone wafer 2 mg.
- Buccal naloxone wafer 4 mg.
- Sublingual naloxone wafer: 2 mg.
- Active comparators:
  - 0.4mg naloxone ampoule (for intramuscular application).
  - 1.8mg intranasal naloxone (Nyxoid®).

Each treatment session is separated by a minimum washout period of 36 hours. Given naloxone’s elimination half-life of approximately 30–80 min, a 36 hour washout represents more than 18 half-lives, rendering pharmacological carryover between sessions negligible.

### Choice of comparators

The 0.4 mg intramuscular naloxone ampoule was selected as the primary reference formulation given its status as the original, most extensively characterised route of naloxone administration, with a well-established PK profile against which novel formulations are typically benchmarked. Intranasal naloxone (Nyxoid®) was included as a secondary reference given its increasing predominance as the preferred formulation for non medically trained administration in take-home naloxone programmes, allowing this study to also generate PK data relevant to the comparator most likely to be encountered in current community practice.

### Setting and participants

The study is conducted at the King’s College London Clinical Research Facility, a purpose-built facility designed for early-phase clinical trials. The facility provides a controlled environment for PK studies with appropriate equipment for blood sampling, vital sign monitoring, and emergency medical support.

### Participant recruitment and enrolment

Participants are recruited through word-of-mouth, university recruitment emails, and approved paper and online advertisements. Interested individuals contact the research team and receive a Participant Information Sheet at least 24 hours before screening. Pre-screening via telephone or video call assesses initial eligibility and allows questions. Participant recruitment started in January 2026 and is currently ongoing. At the time of submission, approximately 50% of the target sample has been recruited and recruitment is expected to be completed by June 2026, with data collection concluding shortly thereafter.

Informed consent is obtained by an authorised physician before any study-specific procedures. Participants are given adequate time to read materials, ask questions, and consider participation. The consent process complies with Good Clinical Practice, the Declaration of Helsinki, and UK regulatory requirements. Participants receive copies of signed consent forms and information sheets.

### Eligibility criteria

Eligible participants are healthy volunteers aged 18–60 years with adequate venous access who are able and willing to provide written informed consent. Healthy status is determined through medical history, physical examination, electrocardiogram, vital signs, and laboratory tests, including full blood count, liver and renal function tests, and urinalysis.

Participants are excluded if they have any clinically significant medical conditions, abnormal screening laboratory values, body mass index outside 18–30 kg/m², blood pressure or heart rate outside normal ranges (90–140/40–90 mmHg; 40–100 beats/minute), estimated glomerular filtration rate ≤70 mL/min, or recent blood donation (>400 mL within 3 months). Medication-related exclusions include any prescribed medications except contraceptives, recent opioid use (within 2–4 weeks, depending on formulation), or positive urine drug screening. Pregnant or breastfeeding women and women of childbearing potential unwilling to use effective contraception are excluded. Additional exclusions include known hypersensitivity to naloxone or formulation excipients (gelatine, mannitol), unwillingness to ingest fish-derived gelatine, alcohol intake >14 units weekly, positive alcohol breath test, participation in another clinical trial within the last 3 months, or insufficient understanding of the trial.

Session-specific exclusions that result in visit postponement rather than study withdrawal include recent opioid use (such as intake of prescribed opioids), positive drug or alcohol screening on the session day, or presence of acute illness, including respiratory symptoms or suspected infection.

### Study procedures

The study follows a standardised protocol across all experimental visits. Participants complete a screening visit (≤28 days prior to first IMP administration), five experimental visits (Stage 1), and a follow-up visit (7–14 days post-final visit, conducted by telephone/online). As seen in the Schedule of Procedures by visit in Fig 1.

**Fig 1:**
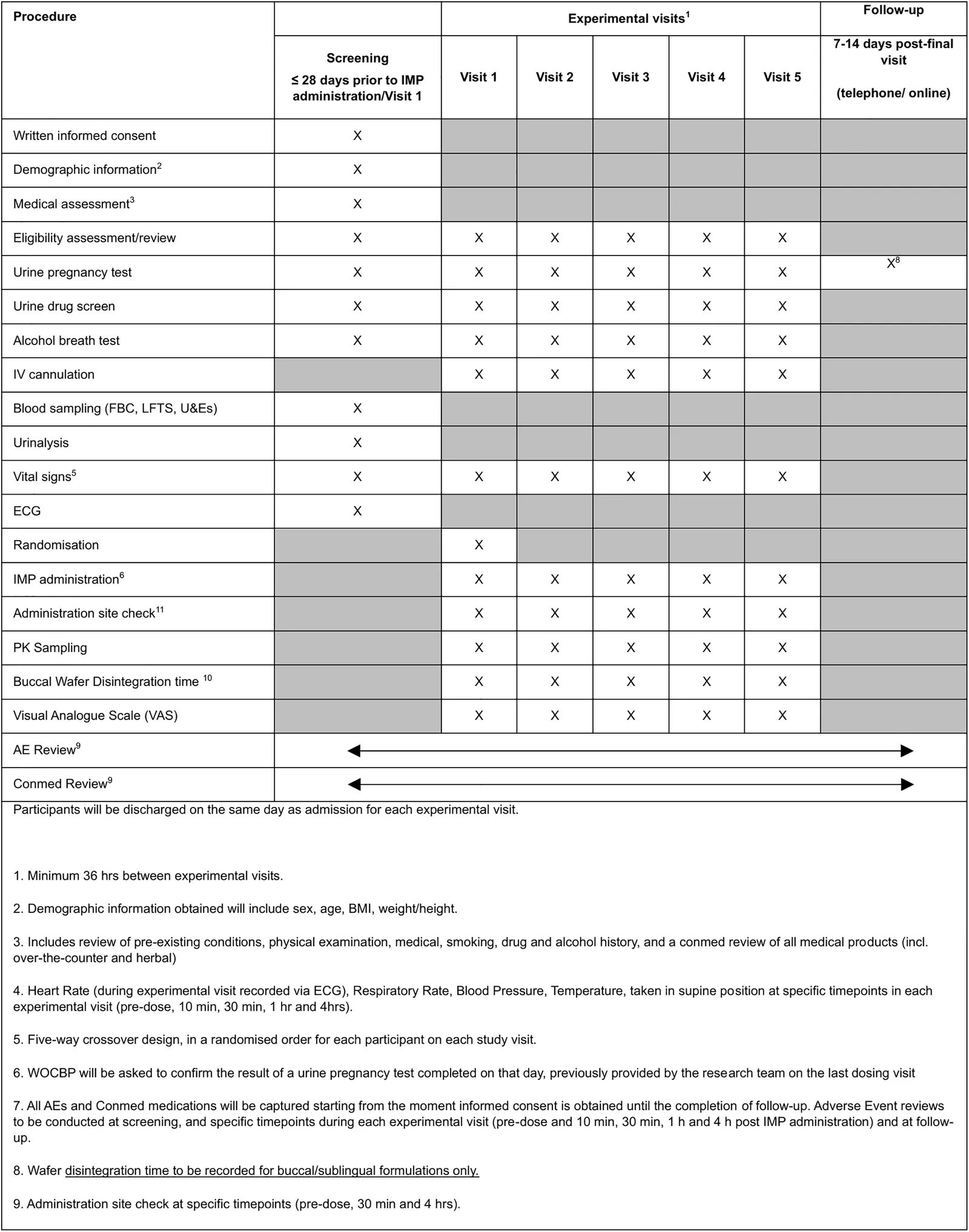
The Schedule of Procedures by visit. The SPIRIT checklist (S1 File) is included as a supplementary file.

#### Screening visit.

At the screening visit, participants undergo:

- Written informed consent
- Demographic information collection
- Medical history and physical examination
- Vital signs (blood pressure, heart rate, respiratory rate, temperature)
- 12-lead electrocardiogram (ECG)
- Height and weight measurement
- Eligibility assessment
- Urine pregnancy test (for women)
- Urine drug screen
- Alcohol breath test
- Blood samples for: full blood count, liver function tests, renal function tests (including estimated glomerular filtration rate).

#### Experimental visits.

Each experimental visit follows an identical protocol with different IMP administration according to the randomisation schedule. Minimum washout period between visits is 36 hours (naloxone half-life <2 hours). A flow diagram of the experimental visits timeline can be seen in more detail in S2File included as a supplementary file.

Visit procedures:

1. Pre-dose assessment (conducted before IMP administration):
  A. Eligibility review and confirmation of continued consent, urine pregnancy test (women), urine drug screen, alcohol breath test, vital signs, concomitant medication review, adverse event assessment, and intravenous cannula insertion
2. IMP administration (time 0):
  A. Participants receive one of five treatments at each experimental visit, according to randomisation:
    i. Novel buccal naloxone wafer 2 mg (placed in cheek pouch)
    ii. Novel buccal naloxone wafer 4 mg (placed in cheek pouch)
    iii. Novel naloxone wafer 2 mg sublingual (placed under tongue)
    iv. Naloxone ampoule 0.4 mg intramuscular
    v. Naloxone intranasal 1.8 mg (Nyxoid®)
3. Post-dose assessment
4. Blood sampling for PK analysis at: 0 (baseline), 2, 4, 6, 8, 10, 12, 15, 20, 30, 45, 60, 90, 120, 180, and 240 minutes post-dose (total 16 timepoints per visit, approximately 6–8 mL per sample)
5. Vital signs at: pre-dose, 10, 30, 60, and 240 minutes post-dose
6. Continuous monitoring and adverse event assessment throughout the 4-hour observation period
7. Cannula removal and final safety check before discharge.

#### Blood sampling schedule.

The frequency and duration of post-dose blood sampling were designed to reflect the time-critical nature of overdose reversal: sampling is dense in the first 30 minutes post-dose (0, 2, 4, 6, 8, 10, 12, 15, 20, 30 minutes) to characterise rapid early absorption and Tmax, tapering to sparser timepoints thereafter (45, 60, 90, 120, 180, 240 minutes). The observation period was extended to 4 hours to assess whether any formulation showed evidence of a longer duration of maintained plasma naloxone levels than existing comparators, which would carry implications for renarcotisation risk in real-world use.

#### Follow-up visit.

A follow-up contact (telephone or online) is conducted 7–14 days after the final experimental visit to assess: adverse events since last visit, concomitant medications, urine pregnancy test (if clinically indicated) and final participant wellbeing check.

#### Randomization.

This study will employ an open-label, randomised, within-subject crossover design with repeated sessions. Each participant will receive all five treatment conditions in a random order, serving as their own control. Sequence order is determined by simple randomisation rather than a structured design such as a Williams design.

As a result, the randomisation schedule may not achieve full balance with respect to treatment order, period, and across the five treatment sequences. Carryover effects between sessions are considered unlikely given the ≥ 36-hour washout period, which exceeds 18 elimination half-lives of naloxone (t½ ~ 1–2 hours). Period and sequence are additionally considered as fixed effects in the mixed-effects model for secondary endpoints to account for any residual order effects.

A web-based randomisation system will be designed, using the bespoke KCTU randomisation system. The randomisation system will be created in collaboration with the trial analyst/s and the CI and maintained by the King’s Clinical Trials Unit for the duration of the project. It will be hosted on a dedicated server within KCL.

Each participant will be assigned a unique study number upon pre-screening, screening and randomisation. The unique randomisation study number allocated to each participant will correspond with the order of treatment for each participant.

#### Blinding.

Treatment allocation is not blinded. Blinding administration route would have required a double-dummy design (placebo buccal/sublingual wafers, placebo intramuscular injections, and placebo intranasal spray at every visit regardless of the active treatment given), adding considerable complexity and participant burden. As the primary and secondary endpoints are objectively measured plasma concentrations rather than participant or observer rated outcomes, the incremental value of blinding was judged not to justify this added burden.

### Study outcomes

#### Primary endpoint.

**Time to Maximum Plasma Concentration (Tmax)**: Time from IMP administration to maximum observed plasma naloxone concentration, measured from baseline to 4 hours post-dose. This endpoint directly addresses the critical need for rapid naloxone absorption during overdose emergencies. A fixed therapeutic plasma concentration threshold for naloxone cannot currently be defined, as the clinically effective concentration depends on the type, potency, and dose of the opioid involved, individual receptor sensitivity, degree of opioid tolerance, and the presence of high-potency synthetic opioids such as fentanyl analogues. In the absence of a validated and universally applicable therapeutic window, Tmax remains the most interpretable primary endpoint for this Phase 1 study. The time to 50% of maximum concentration (T50%) is included as a secondary endpoint to capture clinically relevant early absorption, which may better reflect speed of onset than Tmax alone.

#### Secondary endpoints.

Other PK Parameters:

- **Time to 50% of maximum concentration (T50%):** Time to reach 50% of maximum plasma concentration, measured from baseline to 4 hours post-dose
- **Maximum Plasma Concentration (C**_**max**_**):** Peak plasma naloxone concentration, measured from baseline to 4 hours post-dose
- **Elimination Half-life (T**_1/2_**):** The time required for the plasma concentration of a drug to decrease by 50% during the elimination phase.
- **Area Under the Curve from time zero to 15 minutes (AUC**_0–15_**):** Integral of plasma concentration over time from administration to 15 minutes post-dose, reflecting early drug exposure
- **Area Under the Curve from time zero to infinity (AUC**_**inf**_**):** Total drug exposure calculated from baseline to 4 hours post-dose with extrapolation to infinity.

Safety outcomes:

- Adverse events (AEs) and serious adverse events (SAEs) throughout study participation.
- Vital signs (blood pressure, heart rate, respiratory rate, temperature) at specified timepoints.
- Participant-reported tolerability and acceptability of oral formulation, via Visual Analogue Scale (VAS) Assessment.

Exploratory outcomes:

- Dose-proportionality assessment comparing plasma exposure across different buccal doses
- Comparison of buccal versus sublingual administration of the wafer formulation
- Time to complete wafer disintegration in the buccal cavity.

### Data collection and management

All collected data will be pseudonymised using participant identification codes. Paper Case Report Forms (CRFs) will be completed for each participant at each visit and their data will be securely stored on a secure web-based electronic data capture (EDC) maintained by King’s Clinical Trials Unit.

Blood samples for pharmacokinetic analysis will be processed according to standard operating procedures and will be stored at −80°C until batch analysis. Plasma naloxone concentrations will be measured using a validated liquid chromatography-tandem mass spectrometry (LC-MS/MS) method, conducted by a GLP-compliant, and accredited laboratory. All study data are handled in accordance with Good Clinical Practice (GCP), UK Data Protection Act, and General Data Protection Regulation requirements.

A Trial Steering Committee will be convened independently from the Sponsor for this study and will meet to monitor progress, review safety data, and provide oversight.

### Ethical and safety considerations

The study is conducted in accordance with Good Clinical Practice (GCP) guidelines, the Declaration of Helsinki, and UK regulatory requirements. Ethical approval has been obtained from the London – London Bridge Research Ethics Committee (REC Number: 25/LO/0701; approved 27 October 2025). Clinical Trial Authorisation has been granted by the Medicines and Healthcare products Regulatory Agency (MHRA; IRAS Number: 1011060). The Sponsors for the study are King’s College London and South London and Maudsley NHS Foundation Trust. This trial is registered with the ISRCTN registry (registration number: ISRCTN67173532).

Safety and tolerability are assessed through multiple complementary methods acroos each experimental visit: continuous monitoring and adverse event (AE) assessment, vital signs (blood pressure, heart rate, respiratory rate and temperature) recorded at five timepoints per session (pre-dose, 10, 30 60 and 240 minutes post-dose); and participant-reported tolerability and acceptability via Visual Analogue Scale (VAS). All AEs and serious adverse events (SAEs) are captured from informed consent through to final follow-up and coded using MedDRA terminology.

### Statistical analysis

#### Sample size justification.

A formal sample size calculation has not been performed as this is an exploratory PK study. Given its exploratory objective of generating early pharmacokinetic data to inform the design of a subsequent, adequately powered trial, target enrolment was determined with reference to feasibility and resource constraints rather than a calculated effect size. The minimum of 12 participants aligns with the threshold specified in European Medicines Agency bioequivalence guidance, used here as a practical benchmark rather than as substitute for formal calculation [47]. To account for anticipated attrition across the five experimental visits each participant is expected to attend, recruitment would continue until approximately 15 participants have enrolled. Targeting an estimated 20% dropout rate and preservation of at least 12 participants with complete datasets.

#### Statistical methods.

**PK analysis**: Plasma naloxone concentration-time data will be analysed using non-compartmental methods performed using R (v4.0 or later), an open-source platform. PK parameters (T_max_, C_max_, T_50%_, T_1/2_, AUC_0–15_, AUC_inf_) will be calculated for each participant and treatment arm.

Descriptive statistics (arithmetic mean, standard deviation, coefficient of variation, geometric mean, median, range) will be calculated for all PK parameters by treatment group. Graphical displays will include: Individual and mean plasma concentration-time profiles (linear and semi-logarithmic scales), and individual PK profiles from each treatment session. To visualize within-subject longitudinal shifts across the 5 treatments, subject-specific profile plots will be generated for the raw, untransformed PK parameters (AUC_0–15_, AUC_inf_ and C_max,_ respectively). Each of the 12 participants will be tracked across the treatments via distinct, linked individual lines. To prevent visual clutter while maintaining data transparency, individual lines will be rendered with partial transparency, and the population mean profile will be overlaid as a bold, solid line to clearly depict central tendency alongside individual variability.

For the primary endpoint (T_max_), median and range will be reported. Comparisons between novel formulations and active comparators will be conducted using non-parametric methods (Wilcoxon signed-rank test) with Bonferroni correction for multiple comparisons.

For secondary endpoints (C_max_, AUC_0–15_, AUC_inf_), log-transformed data will be primarily analysed using mixed-effects models with treatment as a fixed effect and a participant-level random intercept. As pre-specified sensitivity analyses, three additional model specifications will be fitted: (1) treatment and period as fixed effects; (2) treatment and sequence as fixed effects; and (3) treatment, period and sequence as fixed effects, each with a participant-level random intercept. Results from all models will be compared. Should the more fully specified models encounter convergence issues or lack of identifiability, given the limited sample size, the period effect will be dropped first; if issues persist, the sequence effect will be dropped instead of period; and if the issues remain, both period and sequence will be omitted, defaulting to the primary treatment-only model. Model fit will be assessed via diagnostic plots of studentized residuals, and results will additionally be presented graphically by subject, sequence, treatment, and period. Geometric mean ratios and 90% confidence intervals will be calculated for novel formulations relative to active comparators.

Dose-proportionality will be assessed by comparing dose-normalised parameters (C_max_/dose, AUC/dose) across buccal doses using power model regression. Buccal versus sublingual administration will be compared using paired statistical tests.

**Safety analysis**: Adverse events will be coded using MedDRA terminology and will be summarised by treatment, severity, and causality. Vital signs will be summarised descriptively at each time point. Clinical laboratory values will be categorised as normal, abnormal but not clinically significant, or abnormal and clinically significant.

**Missing data**: Every effort is made to minimise missing data. Reasons for missing data are documented. No imputation methods are used for PK parameters; analysis is conducted on available data. Sensitivity analyses may be performed if missing data exceeds 10%.

### Protocol amendments and early termination

Any substantive modifications to the study protocol will undergo review and approval by both the Research Ethics Committee (REC) and the study sponsor. The study may be discontinued prematurely if warranted by safety considerations, funding limitations, or administrative decisions, and any such early termination will be reported to the relevant regulatory authorities.

## Discussion

This protocol describes a Phase 1 PK study of a novel ultra-portable buccal naloxone wafer formulation designed to address a critical gap in opioid overdose prevention: the low rate of constant naloxone carriage among individuals at risk. The development of this formulation responds directly to evidence that only 10–20% of people provided with take-home naloxone consistently carry it, primarily due to the impractical features of existing products.

The selection of buccal drug delivery for this formulation is based on several pharmacological and practical advantages. The buccal mucosa provides direct systemic absorption while bypassing first-pass hepatic metabolism, potentially enabling therapeutic plasma concentrations comparable to intravenous administration [40,42,43]. Animal studies have demonstrated 70% bioavailability via buccal administration compared to negligible oral bioavailability, supporting the feasibility of this route [45]. Additionally, buccal administration is simple enough for layperson use, does not require conscious swallowing (clearly important when considering management of overdose), and typically results in rapid mucosal absorption.

Previous successful application of buccal delivery for emergency medications, particularly buccal midazolam for seizure management, demonstrates the viability of this approach for acute medical emergencies [40]. The buccal route has also been used for other medications requiring rapid onset, including sublingual nitro-glycerine and buccal fentanyl, establishing precedent for rapid-acting buccal formulations.

The results of this study will inform the feasibility and optimal formulation for buccal naloxone delivery. If findings demonstrate rapid absorption (T_max_), adequate early drug exposure (AUC _0–15_ comparable to intramuscular administration), and therapeutic plasma concentration (C_max_), these data will support progression to a larger confirmatory study. Conversely, if this study reveals inadequate absorption, the findings will inform whether alternative formulation strategies merit investigation or whether fundamental limitations of buccal delivery for emergency naloxone exist.

Several challenges are anticipated in this research. First, as a PK study in healthy volunteers, it cannot directly assess efficacy in reversing opioid overdose. However, the well-established relationship between naloxone plasma concentrations and opioid receptor antagonism allows PK data to serve as a valid surrogate for therapeutic effect [19]. Second, buccal absorption may exhibit greater inter-individual variability than injectable administration due to differences in buccal mucosa characteristics [48,49]. The crossover design and adequate sample size will help characterize this variability.

Third, the study population of healthy volunteers may not fully represent the target population of people who use opioids, who may have different buccal mucosa characteristics (e.g., due to smoking, dental health, or xerostomia). Future studies in the target population, despite the challenges, would be desirable to confirm PK findings. Fourth, the open-label design may introduce bias in subjective tolerability assessments, though objective PK and safety endpoints are not susceptible to this limitation.

Lastly, the randomisation schedule may not be fully balanced across treatment sequences; residual period and sequence effects, although expected to be minimal, are addressed statistically rather than by design. A Williams design, allocating an initial balanced cohort with additional participants added at random to the same sequence set, would have offered a more formally balanced approach with added capacity for dropout replacement, and we intend to adopt this approach in future studies of this kind.

On the other hand, the buccal wafer formulation seeks to combine the reliability of mucosal administration with the convenience and portability that can enable true constant carriage. If PK parameters prove comparable to or superior to existing formulations, this technology could represent a paradigm shift in harm reduction approaches. A pocket-sized, ambient-stable naloxone product that integrates seamlessly into daily life could dramatically increase carriage rates toward the WHO target of 90% availability at time of future emergency need [50].

The implications extend beyond individual carriage. Employers, schools, public venues, and other settings could more feasibly stock naloxone if it required no special storage and had minimal training requirements for administration. Law enforcement officers, social service providers, and other frontline workers could routinely carry naloxone without burden. Individuals concerned about a friend’s or family member’s opioid use could discreetly keep naloxone available.

Beyond naloxone, this study contributes to a broader understanding of buccal drug delivery for emergency medications. The lyophilisation technology and wafer formulation approach could potentially be applied to other drugs requiring rapid administration in community settings. Success in this application could stimulate innovation in emergency medication delivery systems.

## Supporting information

S1 File SPIRIT Checklist. (DOCX)

S2 File Flow diagram of the experimental visits timeline. (PDF)

S3 File Human Subjects Checklist. (PDF)

S4 File Detailed Protocol. (PDF)

## References

[1] WHO Opioid Overdose: Reducing Preventing and Opioid Overdose Mortality. https://www.who.int/news-room/fact-sheets/detail/opioid-overdose United Nations 2013:1–28.

[2] Office for National Statistics (ONS), released 17 October 2025, ONS website, statistical bulletin, Deaths related to drug poisoning in England and Wales: 2024 registrations

[3] Jordan BA, Cvejic S, Devi LA. Opioids and their complicated receptor complexes. Neuropsychopharmacology. 2000;23(S5):S5–18. doi: 10.1016/S0893-133X(00)00143-3 11008063

[4] Dhaliwal A, Gupta M. Physiology, Opioid Receptor. https://www.ncbi.nlm.nih.gov/books/NBK546642/31536249

[5] Saari TI, Strang J, Dale O. Clinical pharmacokinetics and pharmacodynamics of naloxone. Clin Pharmacokinet. 2024;63:397–422. doi: 10.1007/s40262-024-01355-6 PMC11052794 38485851

[6] E. G. The 1982 John Scott Award goes to Jack Fishman and Harold Blumberg for synthesis and investigations on naloxone. Essay Inf Sci. 1983;:121–30.

[7] Pasternak GW, Pan Y-X. Mu opioids and their receptors: evolution of a concept. Pharmacol Rev. 2013;65(4):1257–317. doi: 10.1124/pr.112.007138 24076545 PMC3799236

[8] Trøstheim M, Eikemo M, Haaker J, Frost JJ, Leknes S. Opioid antagonism in humans: a primer on optimal dose and timing for central mu-opioid receptor blockade. Neuropsychopharmacology. 2023;48(2):299–307. doi: 10.1038/s41386-022-01416-z 35978096 PMC7613944

[9] Strang J, McDonald R. Preventing opioid overdose deaths with take-home naloxone. 2016.

[10] McDonald R, Campbell ND, Strang J. Twenty years of take-home naloxone for the prevention of overdose deaths from heroin and other opioids-Conception and maturation. Drug Alcohol Depend. 2017;178:176–87. doi: 10.1016/j.drugalcdep.2017.05.001 28654870

[11] WHO Community Management of Opioid Overdose. 2014. 25577941

[12] McDonald R, Strang J. Are take-home naloxone programmes effective? Systematic review utilizing application of the Bradford Hill criteria. Addiction. 2016;111(8):1177–87. doi: 10.1111/add.13326 PMC5071734 27028542

[13] Strang J, McDonald R, Campbell G, Degenhardt L, Nielsen S, Ritter A, et al. Take-Home Naloxone for the Emergency Interim Management of Opioid Overdose: The Public Health Application of an Emergency Medicine. Drugs. 2019;79(13):1395–418. doi: 10.1007/s40265-019-01154-5 31352603 PMC6728289

[14] Ko J, Chan E, Doroudgar S. Patient perspectives of barriers to naloxone obtainment and use in a primary care, underserved setting: A qualitative study. Subst Abus. 2021;42(4):1030–9. doi: 10.1080/08897077.2021.1915915 33945451

[15] Bessen S, Metcalf SA, Saunders EC, Moore SK, Meier A, McLeman B, et al. Barriers to Naloxone Use and Acceptance among Opioid Users, First Responders, and Emergency Department Providers in New Hampshire, USA. Int J Drug Policy. 2019;74:144–51. doi: 10.1016/j.drugpo.2019.09.008 PMC7153573 31590090

[16] Kozak Z, Ciccarone D, Thrul J, Cole TO, Pappas AL, Greenblatt AD, et al. Harm reduction behaviors are associated with carrying naloxone among patients on methadone treatment. Harm Reduct J. 2023;20(1):17. doi: 10.1186/s12954-023-00745-6 36788601 PMC9928141

[17] Pellissier G, Miguéres B, Tarantola A, Abiteboul D, Lolom I, Bouvet E, et al. Risk of needlestick injuries by injection pens. J Hosp Infect. 2006;63(1):60–4. doi: 10.1016/j.jhin.2005.12.006 16540200

[18] Strang J, McDonald R, Alqurshi A, Royall P, Taylor D, Forbes B. Naloxone without the needle - systematic review of candidate routes for non-injectable naloxone for opioid overdose reversal. Drug Alcohol Depend. 2016;163:16–23. doi: 10.1016/j.drugalcdep.2016.02.042 26996745

[19] Rzasa Lynn R, Galinkin JL. Naloxone dosage for opioid reversal: current evidence and clinical implications. Ther Adv Drug Saf. 2018;9(1):63–88. doi: 10.1177/2042098617744161 29318006 PMC5753997

[20] Doe-Simkins M, Walley AY, Epstein A, Moyer P. Saved by the nose: bystander-administered intranasal naloxone hydrochloride for opioid overdose. Am J Public Health. 2009;99(5):788–91. doi: 10.2105/AJPH.2008.146647 19363214 PMC2667836

[21] EUDA Take-Home Naloxone. https://www.euda.europa.eu/publications/topic-overviews/take-home-naloxone_en

[22] McDonald R, Parkin S, Eide D, Neale J, Clausen T, Metrebian N, et al. Rethinking “carriage” of take-home naloxone. Int J Drug Policy. 2021;95:103253. doi: 10.1016/j.drugpo.2021.103253 33848942

[23] Strang J, Darke S, Hall W, Farrell M, Ali R. Heroin overdose: the case for take-home naloxone. BMJ. 1996;312(7044):1435–6. doi: 10.1136/bmj.312.7044.1435 8664611 PMC2351168

[24] Chung S, Peters JM, Detyniecki K, Tatum W, Rabinowicz AL, Carrazana E. The nose has it: Opportunities and challenges for intranasal drug administration for neurologic conditions including seizure clusters. Epilepsy Behav Rep. 2022;21:100581. doi: 10.1016/j.ebr.2022.100581 36636458 PMC9829802

[25] Jiao J, Zhang L. Influence of Intranasal Drugs on Human Nasal Mucociliary Clearance and Ciliary Beat Frequency. Allergy Asthma Immunol Res. 2019;11(3):306–19. doi: 10.4168/aair.2019.11.3.306 30912321 PMC6439188

[26] Dahan A, Franko TS, Carroll JW, Craig DS, Crow C, Galinkin JL, et al. Fact vs. fiction: naloxone in the treatment of opioid-induced respiratory depression in the current era of synthetic opioids. Front Public Health. 2024;12:1346109. doi: 10.3389/fpubh.2024.1346109 38481848 PMC10933112

[27] Gonzalez Utrilla M, Chesney E, Neale J, Metrebian N, Kalk N, Skulberg AK, et al. Naloxone dosing in the era of synthetic opioids: Applying the Goldilocks principle. Addiction. 2025;120(11):2165–72. doi: 10.1111/add.70060 40197810 PMC12529241

[28] Van Arsdale W, Barajas S, Peters D, Marques O, Sacca L. Is naloxone where it needs to be? Using spatial analytics to examine equitable distribution of community-based naloxone sites. Journal of Substance Use and Addiction Treatment. 2025;168:209530. doi: 10.1016/j.josat.2024.209530 39389545

[29] Dernbach MR, Dasgupta N, Sibley AL, Tylleskar I, Skulberg AK. A chain of survival for drug overdose. Resusc Plus. 2025;25:101026. doi: 10.1016/j.resplu.2025.101026 40703815 PMC12284353

[30] Cooper-Ohm S, Habecker P, Humeniuk R, Bevins RA. Factors Associated with Gaps in Naloxone Knowledge: Evidence from a 2022 Great Plains Survey. Res Sq. 2023;:rs.3.rs-3536993. doi: 10.21203/rs.3.rs-3536993/v1 38336722 PMC10858634

[31] Scotland PH, University GC, Centre W of SSV. The needle exchange surveillance initiative (NESI): Monitoring blood-borne viruses and injecting risk behaviours among people who inject drugs in Scotland, 2008-09 to 2022-23. 2024.

[32] Amaducci A, Aldy K, Campleman SL, Li S, Meyn A, Abston S, et al. Naloxone Use in Novel Potent Opioid and Fentanyl Overdoses in Emergency Department Patients. JAMA Netw Open. 2023;6(8):e2331264. doi: 10.1001/jamanetworkopen.2023.31264 37642962 PMC10466160

[33] Skulberg AK, Tylleskar I, Nilsen T, Skarra S, Salvesen Ø, Sand T, et al. Pharmacokinetics and -dynamics of intramuscular and intranasal naloxone: an explorative study in healthy volunteers. Eur J Clin Pharmacol. 2018;74(7):873–83. doi: 10.1007/s00228-018-2443-3 29568976

[34] McDonald R, Lorch U, Woodward J, Bosse B, Dooner H, Mundin G, et al. Pharmacokinetics of concentrated naloxone nasal spray for opioid overdose reversal: Phase I healthy volunteer study. Addiction. 2018;113(3):484–93. doi: 10.1111/add.14033 29143400 PMC5836974

[35] Mundin G, McDonald R, Smith K, Harris S, Strang J. Pharmacokinetics of concentrated naloxone nasal spray over first 30 minutes post-dosing: analysis of suitability for opioid overdose reversal. Addiction. 2017;112:1647–52. doi: 10.1111/add.13849 28430384

[36] Jolley CJ, Bell J, Rafferty GF, Moxham J, Strang J. Understanding Heroin Overdose: A Study of the Acute Respiratory Depressant Effects of Injected Pharmaceutical Heroin. PLoS One. 2015;10(10):e0140995. doi: 10.1371/journal.pone.0140995 26495843 PMC4619694

[37] Yassen A, Olofsen E, van Dorp E, Sarton E, Teppema L, Danhof M, et al. Mechanism-based pharmacokinetic-pharmacodynamic modelling of the reversal of buprenorphine-induced respiratory depression by naloxone: a study in healthy volunteers. Clin Pharmacokinet. 2007;46(11):965–80. doi: 10.2165/00003088-200746110-00004 17922561

[38] Ryan SA, Dunne RB. Pharmacokinetic properties of intranasal and injectable formulations of naloxone for community use: a systematic review. Pain Manag. 2018;8(3):231–45. doi: 10.2217/pmt-2017-0060 29683378

[39] Dietze P, Jauncey M, Salmon A, Mohebbi M, Latimer J, van Beek I, et al. Effect of Intranasal vs Intramuscular Naloxone on Opioid Overdose: A Randomized Clinical Trial. JAMA Netw Open. 2019;2(11):e1914977. doi: 10.1001/jamanetworkopen.2019.14977 31722024 PMC6902775

[40] McIntyre J, Robertson S, Norris E, Appleton R, Whitehouse WP, Phillips B, et al. Safety and efficacy of buccal midazolam versus rectal diazepam for emergency treatment of seizures in children: a randomised controlled trial. Lancet. 2005;366(9481):205–10. doi: 10.1016/S0140-6736(05)66909-7 16023510

[41] Alqurshi A, Kumar Z, McDonald R, Strang J, Buanz A, Ahmed S, et al. Amorphous Formulation and in Vitro Performance Testing of Instantly Disintegrating Buccal Tablets for the Emergency Delivery of Naloxone. Mol Pharm. 2016;13(5):1688–98. doi: 10.1021/acs.molpharmaceut.6b00096 26977787

[42] Sudhakar Y, Kuotsu K, Bandyopadhyay AK. Buccal bioadhesive drug delivery - a promising option for orally less efficient drugs. Journal of Controlled Release. 2006;114:15–40. doi: 10.1016/j.jconrel.2006.04.012 16828915

[43] Pather SI, Rathbone MJ, Senel S. Current status and the future of buccal drug delivery systems. Expert Opin Drug Deliv. 2008;5(5):531–42. doi: 10.1517/17425247.5.5.531 18491980

[44] Sattar M, Sayed OM, Lane ME. Oral transmucosal drug delivery - current status and future prospects. Int J Pharm. 2014;471:498–506. doi: 10.1016/j.ijpharm.2014.05.043 24879936

[45] Hussain MA, Aungst BJ, Kearney A, Shefter E. Buccal and oral bioavailability of naloxone and naltrexone in rats. Int J Pharm. 1987;36:127–30. doi: 10.1016/0378-5173(87)90147-5

[46] Mazzinelli E, Favuzzi I, Arcovito A, Castagnola R, Fratocchi G, Mordente A, et al. Oral Mucosa Models to Evaluate Drug Permeability. Pharmaceutics. 2023;15(5):1559. doi: 10.3390/pharmaceutics15051559 37242801 PMC10220859

[47] European Medicines Agency, C. for M.P. for H.U. (CHMP) Guideline on the Investigation of Bioequivalence (Rev. 1); 2010. 10.1111/j.1742-7843.2009.00518.x 20070293

[48] Bartlett JA, van der Voort Maarschalk K. Understanding the oral mucosal absorption and resulting clinical pharmacokinetics of asenapine. AAPS PharmSciTech. 2012;13(4):1110–5. doi: 10.1208/s12249-012-9839-7 22936407 PMC3513449

[49] Davies A, Finn A, Tagarro I. Intra- and interindividual variabilities in the pharmacokinetics of fentanyl buccal soluble film in healthy subjects: a cross-study analysis. Clin Drug Investig. 2011;31(5):317–24. doi: 10.1007/BF03256930 21294598

[50] World Health Organization. Opioid Overdose. https://www.who.int/news-room/fact-sheets/detail/opioid-overdose Accessed 2023 October 1.

