# Supplementary material for: Buccal and sublingual lyophilised naloxone for opioid overdose reversal: A Phase 1 open-label, random-sequence crossover pharmacokinetic study protocol in healthy volunteers (NalBuc)
Source: PLoS One. 2026 Oct 27;21(10):e0347370. doi: 10.1371/journal.pone.0347370 (PMC13712621; doi:10.1371/journal.pone.0347370)
Supplement: Supplementary file 1 [file pone.0347370.s002.pdf]

S2. Assessment Timepoints for each of the Experimental Visits in Stage 1

Assessment	Timepoint																
	Pre-dose		Post-dose														
	Pre	-5m	0m	2m	4m	6m	8m	10m	12.5m	15m	30m	45m	1h	1.5h	2h	4h	> 4h
Eligibility assessment/review	X																
Urine pregnancy	X																
Urine drug screen	X																
Alcohol breath test	X																
IV cannulation	X																
Vital sign assessments[1]	X							X			X		X			X	
Treatment allocation/ randomisation[2]	X																
IMP administration[3]			X														
Administration site check[4]	X										X					X	
PK blood sampling		X		X	X	X	X	X	X	X	X	X	X	X	X	X	
Wafer disintegration time[5]			X														
Visual Analogue Scale (VAS)[6]			X														
Discharge																	X[7]
AE Review[8]		X						X			X		X			X	
Conmed review	←——————————————————————————————→																

In this five-way crossover design study in stage 1, all treatment experimental visits will follow this schedule.

1. Vital sign assessments include. Heart rate, respiratory rate, blood pressure, and oral body temperature.
2. Each participant will be assigned a randomised treatment allocation sequence for drug administration ordering either at or before visit 1, pre-dose.
3. IMP administration is allocated according to the randomised treatment allocation ordering, determined at visit 1, pre-dose.

4. For buccal and sublingual treatment arms, oral mucosa will be examined with any changes documented. IM injection site and nasal cavities will be examined in the other arms.
5. Wafer disintegration time only recorded for buccal and sublingual naloxone administration visits.
6. VAS assessment completed by participants immediately following administration of each oral formulation to assess taste and tolerability.
7. Discharge at least 4 hours post dose at minimum.
8. AE review reported at anytime, however participants will be prompted by research staff at these timepoints.
