# Supplementary material for: Buccal and sublingual lyophilised naloxone for opioid overdose reversal: A Phase 1 open-label, random-sequence crossover pharmacokinetic study protocol in healthy volunteers (NalBuc)
Source: PLoS One. 2026 Oct 27;21(10):e0347370. doi: 10.1371/journal.pone.0347370 (PMC13712621; doi:10.1371/journal.pone.0347370)
Supplement: Supplementary file 3 [file pone.0347370.s004.pdf]

PROTOCOL FULL TITLE: ULTRA-PORTABLE RAPID-DISPERSAL BUCCAL LYOPHILISED NALOXONE FOR CONSTANT CARRIAGE: TESTING IN HEALTHY VOLUNTEERS

Protocol Short Title/ Acronym: Buccal naloxone testing in healthy volunteers (NalBuc)

Version: 2.0 24/OCT/2025

Trial Identifiers

REC Number:	25/LO/0701
IRAS Number:	1011060
Other Trial Identifiers:	3644NalBuc

(Co) Sponsor(s)

Name of organisation(s):	King's College London<br>South London and Maudsley NHS Foundation Trust
(Co)Sponsor(s') contact:	Ann-Marie Murtagh
Address:	King's Health Partners Clinical Trials Office, Floor 16, Tower Wing, Guy's Hospital, Great Maze Pond, London, SE1 9RT
Telephone:	020 71885732
Email:	QM.KHPCTO@kcl.ac.uk

Chief Investigator

Name:	Professor Sir John Strang
Address:	4 Windsor Walk, Denmark Hill, London SE5 8BB
Telephone:	0207 848 0438
Mobile:	07969 587046
Email:	john.strang@kcl.ac.uk

Name and address of Co-Investigators, Statisticians, CTU

Co-Investigators

Name:	Mariana Gonzalez Utrilla
Address:	4 Windsor Walk, Denmark Hill, London SE5 8BB
Telephone:	0207 848 0438
Email:	mariana.gonzalez_utrilla@kcl.ac.uk

Name:	Dr Edward Chesney
Address:	4 Windsor Walk, Denmark Hill, London SE5 8BB
Telephone:	0207 848 0438
Email:	Edward.chesney@kcl.ac.uk

Name:	Alexander Baker
Address:	4 Windsor Walk, Denmark Hill, London SE5 8BB
Telephone:	0207 848 0438
Email:	Alexander.s.baker@kcl.ac.uk

Name:	Dr Elizabeth Appiah-Kusi
Address:	4 Windsor Walk, Denmark Hill, London SE5 8BB
Telephone:	0207 848 0438
Email:	elizabeth.appiah-kusi@kcl.ac.uk

Name:	Dr Martine Skumlien
Address:	4 Windsor Walk, Denmark Hill, London SE5 8BB
Telephone:	0207 848 0438
Email:	martine.skumlien@kcl.ac.uk

Statisticians

Name:	Kim Lee (NIHR Maudsley BRC)
Address:	16 De Crespigny Park, London, SE5 8AF, United Kingdom
Telephone:	0207 848 0724
Email:	kim.lee@kcl.ac.uk

Name:	Richard Emsley (NIHR Maudsley BRC)
Address:	NIHR Maudsley BRC, Denmark Hill, London, SE5 8AF
Telephone:	0207 848 0724

Email:	richard.emsley@kcl.ac.uk

Laboratories

Name:	Synnovis
Address:	King's College Hospital, Bessemer Wing, Denmark Hill, London SE5 9RS
Telephone:	Contracts Team: 020 3299 4144; Scientific Team: 020 3229 5548
Email:	Contracts Team: kch-tr.synnovis.researchenquiries@nhs.net<br>Scientific Team: kch-tr.contractrandd@nhs.net

Name:	PK Laboratory - John Halket & Anna Caldwell
Address:	Department of Nutritional Sciences, Franklin-Wilkins Building, 150 Stamford Street, London SE1 9NH
Email:	john.halket@kcl.ac.uk<br>anna.caldwell@kcl.ac.uk

King’s Clinical Trials Unit

Name:	King’s Clinical Trials Unit
Address:	King’s College London PO64, Room M2.06, Institute of Psychiatry, 16 De Crespigny Park, London, SE5 8AF
Telephone:	020 7848 0532
Email:	ctu@kcl.ac.uk

1. Study Synopsis

Title of clinical trial	Ultra-portable rapid-dispersal buccal lyophilised naloxone for constant carriage: testing in healthy volunteers
Protocol Short Title/Acronym	Buccal naloxone testing in healthy volunteers (NalBuc)
Trial Phase	Phase 1 Pharmacokinetic Study
(Co-)-Sponsor(s) name	King's College London and South London and Maudsley NHS Foundation Trust
Chief Investigator	Professor Sir John Strang
IRAS number	1011060
REC number	25/LO/0701
Medical condition or disease under investigation	Opioid overdose
Purpose of clinical trial	To study the absorption of different formulations of naloxone in healthy volunteers who will receive both this novel buccal naloxone wafer versus current forms of naloxone, with attention to the speed of absorption and best dose for future medicinal products.
Primary objective	To characterize and compare the pharmacokinetic profile of a novel buccal formulation of naloxone in healthy volunteers with approved formulations (naloxone ampoule) as the injectable form, and Nyxoid® as naloxone nasal spray comparison.
Secondary objective (s)	1. To measure the dose-proportionality of the novel formulation by comparing the plasma exposure of two different buccal doses and one sublingually.<br>2. To assess the safety and tolerability of the novel buccal formulation
Trial design	Phase 1 CTIMP.<br>Pharmacokinetic study.<br>Open-label, within-subject, repeated-sessions, random sequence, two-stage design.<br>Stage 1 uses a five-way crossover design for treatment selection.

	Stage 2 uses another crossover design when at least one of the three novel formulations of naloxone meets the treatment selection criteria, otherwise, the study will be terminated.
Primary Endpoint	Time to Maximum Plasma Concentration - T_{max} measured from baseline to 4 hours post.
Secondary Endpoints	Time to 50% of maximum concentration -T50%, measured from baseline to 4 hours post.<br>Maximum Plasma Concentration - $C_{max,}$ measured from baseline to 4 hours post.<br>Elimination Half-life - $T_{1/2}$ measured from baseline to 4 hours post.<br>Area Under the Curve from time zero to 15 minutes, measured from baseline to 15 minutes post-dose.<br>Area Under the Curve from time zero to infinity - AUC_{inf} measured from baseline to 4 hours post.
Sample size	12 participants in Stage 1.<br>30-35 participants in Stage 2.<br>Participants that partake in stage 1 will not be eligible to also participate in stage 2.
Summary of eligibility criteria	**Inclusion criteria**<br>I.01. Healthy volunteers. Defined as healthy based on a clinical history, physical examination, ECG, vital signs, and laboratory tests of blood and urine.<br>I.02. Age 18-60<br>I.03. Able and willing to provide written informed consent<br>I.04. Adequate venous access and willingness for intravenous cannulation during each visit.<br><br>**Exclusion criteria**<br>E.01. Clinically relevant medical history, physical findings, ECG, or laboratory values at the pre-trial screening assessment that could interfere with the objectives of the trial or the safety of the participant.<br>E.02. Presence of acute or chronic illness or history of chronic illness sufficient to invalidate the volunteer's participation in the trial or make it unnecessarily hazardous.<br>E.03. Impaired endocrine, thyroid, hepatic, respiratory or renal function, diabetes mellitus, coronary heart disease, or history of any neurological or mental illness.

	E.04. Surgery or medical condition that might affect the absorption of medicines.<br>E.05. Blood pressure and heart rate in the supine position at the screening examination outside the ranges: blood pressure 90–140 mm Hg systolic, 40–90 mm Hg diastolic; heart rate 40–100 beats/min. Repeat measurements are permitted if values are borderline (i.e. values that are within 5 mm Hg for blood pressure or 5 beats/min for heart rate) or if requested by the investigator. Subjects can be included if the repeat value is within range or still borderline but deemed not clinically significant by the investigator.<br>E.06. Loss of more than 400 mL of blood during the 3 months before the trial, e.g. as a blood donor.<br>E.07. Any prescribed medication (apart from contraceptives).<br>E.08. Use of any over-the-counter medications containing codeine or other opioids, prescribed opioid medication, or illicitly obtained opioids within the past 2 weeks (if the participant is taking a long-acting opioid the period might, after consideration by the examining doctor, be extended to 4 weeks or longer according to the washout period).<br>E.09. BMI <18 or >30.0kg/m2.<br>E.10. Intake of more than 14 units of alcohol weekly.<br>E.11. Pregnant or breastfeeding.<br>E.12. Women of childbearing potential (as defined in CTFG guidelines, see 6.7 Concomitant Medication) not willing to use a highly effective form of contraception (as defined in CTFG guidelines, see section 6.7 Concomitant Medication) during participation in the study or male patients not willing to ensure the use of a condom during participation in the study.<br>E.13. eGFR≤ 70 ml/min.<br>E.14. Any liver function or renal function test abnormality. A repeat is allowed on one occasion for determination of eligibility.<br>E.15. Urine drug screen positive for any substances.<br>E.16. Positive alcohol breath test, above 0.

	E.17. Participant in any other clinical trial or experimental drug study in the past 3 months<br>E.18. Known hypersensitivity to naloxone and/or formulation excipients (gelatin, mannitol).<br>E.19. Not willing to ingest fish-derived gelatin.<br>E.20. Insufficient understanding of the trial.
IMP, dosage and route of administration	A novel rapid-dispersal ultra-portable formulation of naloxone-at two test doses (2mg and 4mg) administered for buccal absorption in the cheek pouch, and a 2 mg dose administered sublingually.<br>After the initial 12 participants in stage 1, IMP will be compared to active comparators to ensure relevance and competitiveness. IMP treatment arms may be discontinued for stage 2 following this review.
Active comparator product(s)	Naloxone ampoule of 0.4mg/1mL<br>Intranasal naloxone 1.8mg/0.1mL (Nyxoid®)
Maximum duration of treatment of a participant	Single dose/arm<br>Maximum three months duration/participant (expected)

2. Protocol Version History

Version Number	Date	Reasons for Update
2.0	24 OCT 2025	Updates requested by MHRA/REC via GNA response

3. Glossary of Terms

AE	Adverse Event
AR	Adverse Reaction
AUC_{0-15}	Area Under the Curve from time zero to 15 minutes
AUC_{inf}	Area Under the Curve from time zero to infinity
BP	Blood Pressure
CA	Competent Authority
CI	Chief Investigator
Cmax	Maximum Plasma Concentration
CRA	Clinical Research Associate
CRF	Case Report Form
CRF	Clinical Research Facility
CTA	Clinical Trial Authorisation
CTIMP	Clinical Trial of Investigational Medicinal Product
DSUR	Development Safety Update Report
EC	European Commission
EU	European Union
FBC	Full Blood Count
FDA	Food and Drug Administration
FSH	Follicle-stimulating Hormone
GCP	Good Clinical Practice
HR	Heart Rate
IB	Investigator Brochure
ICF	Informed Consent Form
IM	Intramuscular
IMP	Investigational Medicinal Product
IMPD	Investigational Medicinal Product Dossier
IN	Intranasal
IUD	Intrauterine Device
IUS	Intrauterine Hormone-releasing System
IV	Intravenous
LFTs	Liver Function Tests

MA	Marketing Authorisation
MHRA	Medicines and Healthcare products Regulatory Agency
MOR	Mu-Opioid Receptor
NIMP	Non-Investigational Medicinal Product
PI	Principal Investigator
PIS	Participant Information Sheet
QA	Quality Assurance
QP	Qualified Person
REC	Research Ethics Committee
SAE	Serious Adverse Event
SAR	Serious Adverse Reaction
SLaM	South London and Maudsley NHS Foundation Trust
SC	Subcutaneous
SmPC	Summary of Product Characteristics
SOP	Standard Operating Procedure
SUSAR	Suspected Unexpected Serious Adverse Reaction
T1/2	Elimination Half-life
T50%	Time to 50% of maximum concentration
Temp	Temperature
Tmax	Time to Maximum Plasma Concentration
TMF	Trial Master File
TMG	Trial Management Group
TSC	Trial Steering Committee
UAR	Unexpected Adverse Reaction
U&E	Urea & Electrolytes
VAS	Visual Analogue Scale
WOCBP	Woman of Childbearing Potential

CONTENTS

1. Study Synopsis 4
2. Protocol Version History 8
3. Glossary of Terms 8
4. Introduction 12
4.1. Background & Rationale 12
4.2. Scientific Rationale 14
5. Trial Objectives and Design 15
5.1. Trial Objectives 15
5.2. Primary endpoints 15
5.3. Secondary endpoints 15
5.4. Trial Design 16
5.5. Trial Flowchart 17
6. Trial Medication 18
6.1. Investigational Medicinal Product 18
6.2. Dosing Regimen 20
6.3. IMP Risks 21
6.4. Drug Accountability 22
6.5. Storage of IMP 23
6.6. Participant Compliance 23
6.7. Concomitant Medication 23
7. Selection and Withdrawal of Participants 24
7.1. Inclusion Criteria 24
7.2. Exclusion Criteria 24
7.3. Selection of Participants 25
7.4. Consent 25
7.5. Randomisation Procedure / Code Break 26
7.6. Withdrawal of Participants 27
7.7. Expected Duration of Trial 28
8. Trial Procedures 28
8.1. Study Schedule of Assessments 29
8.2. By Visit 30
8.3. Laboratory Tests 31
9. Assessment of Efficacy 33
9.1. Efficacy Parameters 33
9.2. Procedures for Assessing Efficacy Parameters 33
10. Assessment of Safety 34
10.1. Specification, Timing and Recording of Safety Parameters 34
10.2. Procedures for Recording and Reporting Adverse Events 35
10.3. Adverse events that do not require reporting 36
10.4. Premature Termination of the Trial 36
11. Statistics 37
11.1. Sample Size 37

11.2. Randomisation ... 37
11.3. Analysis ... 37
12. Trial Management Group (TMG) ... 39
13. Trial Steering Committee ... 39
14. Direct Access to Source Data and Documents ... 39
15. Ethics & Regulatory Approvals ... 39
16. Quality Assurance ... 40
17. Data Management ... 40
17.1. Access to the final trial dataset ... 41
17.2. Data management in the event of early withdrawal ... 41
18. Publication Policy ... 41
19. Insurance / Indemnity ... 41
20. Financial Aspects ... 41
20.1. Payment ... 42
21. Archiving ... 42
22. Signatures ... 42
REFERENCES ... 43

LIST OF TABLES

Table 1 Clinical Trial Plan by Visit ... 17
Table 2 Marketing authorisation numbers of IMP ... 19
Table 3 Adverse reactions for naloxone ... 22
Table 4 Treatment Coding for Randomisation ... 26
Table 5 Assessment Timepoints for each of the Experimental Visits in Stage 1 ... 29
Table 6 Laboratory Tests ... 32

4. Introduction

4.1. Background & Rationale

Opioids continue to be the substances with the highest contribution to severe drug-related harm, including fatal overdoses [1]. Opioid overdoses account for an estimated 150,000 global deaths annually and approximately 3,000 across the UK, representing a significant public health crisis [2]. Opioids act on the central nervous system to stimulate opioid receptors in the brain and body, which intercept pain signals. [3] However, they can also cause respiratory depression, which in cases of overdose can lead to hypoxia, brain damage, and death [4]. Opioid overdose is both preventable and if witnessed, treatable (reversible) [1].

Naloxone is a potent opioid antagonist that rapidly reverses the effects of opioid overdose [4]. It actively displaces opioids from the mu-opioid receptor (MOR), effectively displacing and reversing their effects, particularly respiratory depression [5]. Naloxone in injectable form has been used in clinical settings since the 1970s and has a well-established safety profile [6]. Its effectiveness in reversing opioid overdose is well-documented, with the potential to restore normal breathing within 2-3 minutes of administration [7,8].

The US Food and Drug Administration (FDA) approved naloxone in 1971 as a prescription - only medication for intravenous (IV), intramuscular (IM), and subcutaneous (SC) administration to reverse the effects of opioids [9,10]. It was subsequently adopted in international clinical practice and included in the World Health Organization's Model List of Essential Medicines in 1983 as a specific antidote (0.4 mg in 1mL ampoules) and has been continuously available across the UK and many other countries in injectable forms since the early 1980s [1,11]. Due to its unique effectiveness and safety profile, naloxone has become the preferred treatment for reversing opioid overdoses in hospital emergency departments and ambulance services [10].

However, the life-saving potential of naloxone is critically dependent on its availability and prompt administration during an overdose event [12]. Recognizing this, many countries have implemented take-home naloxone programs, providing the medication to individuals at risk of overdose, their friends and family members, and community workers. These programs have shown promising results in reducing opioid overdose mortality [13,14].

Nonetheless, the use of injections in emergency situations is limited by public and political apprehension about the wider use of injectable medication as well as the training required for proper administration and the lack of portability of syringes [15]. Therefore, alternative non-injectable options need to be considered [8,16]. More recently, since 2017, nasal sprays have been introduced, offering a needle-free option that is easier for lay persons to administer [6,17]. The main forms currently in the UK are 'Prenoxad' (pre-filled syringe, 5 x 0.4mg doses) and 'Nyxoid®' (naloxone 1.8mg/0.1mL nasal spray, twin-pack) and, recently, generic nasal spray (1.26mg, also twin-pack) [18,19]. However, these products are not convenient for constant carriage and have limited flexibility of dose which may prove insufficient for future overdose crisis situations [20]. Similarly, their effectiveness can be compromised in cases where nasal passages are blocked or damaged [8].

Despite these options, significant barriers persist with the sought-after benefit from naloxone pre-provision [8,21,22]. A key issue is the low carriage rate, with studies indicating that only 15-20% of those provided with naloxone consistently carry it [23,24]. This low rate significantly reduces the likelihood of naloxone being available when an overdose occurs [20].

This study will evaluate a novel buccal formulation of lyophilised naloxone in the form of rapid-dispersal wafers. This innovative approach aims to address the shortcomings of existing

naloxone formulations by improving portability whilst maintaining, crucially, the speed of drug absorption and duration of action.

4.1.1. Rationale for Dose

The selection of 2mg and 4mg doses for this study is primarily based on evidence from transmucosal naloxone delivery. The proposed 2mg dose represents our starting point and this is based primarily on the established intranasal naloxone formulations which are already approved in UK, across Europe and in Australia. Nyxoid, the first approved naloxone nasal spray, contains 1.8mg naloxone hydrochloride dihydrate (equivalent to 2mg naloxone hydrochloride) and this achieves plasma concentrations over the first 10 minutes that are comparable to the standard 0.4mg intramuscular dose [25,26]. Nasal mucosa was chosen for the nasal sprays because it is highly vascularised tissue that permits direct systemic absorption, bypassing hepatic first-pass metabolism, and the same applies to the oral mucosa which is relevant to the buccal and sublingual routes of administration which are now proposed to be tested. The venous drainage from the buccal mucosa flows directly into the internal jugular vein via the retromandibular, lingual, and facial veins, providing rapid systemic delivery [27,28].

Direct human pharmacokinetic data for buccal naloxone are not yet available (and will be produced by the proposed study). Animal studies have been conducted: study in rats demonstrated approximately 70% bioavailability from buccal administration compared to only 0.3% via the oral route, with maximum plasma levels achieved within 15 minutes [29]; and a more recent study in dogs found time to maximum plasma concentrations at 15 minutes and 18 minutes with two different doses of buccal nasal swab, compared with 22 minutes with intramuscular naloxone, and with bioavailability relative to intramuscular at 36% and at 25% for the two different buccal dosages studied [30].

The inclusion of the 4mg dose serves multiple scientific and clinical purposes. First, it allows us to partially assess dose-proportionality of the novel formulation, a critical pharmacokinetic parameter for future dose optimisation. Second, current naloxone nasal spray products are already supplied as twin-packs (Nyxoid 1.8mg/0.1mL and generic naloxone 1.26mg/0.1mL nasal sprays in the UK and across Europe; Narcan 4mg/0.1mL in the US and Canada), with clinical guidance often recommending administration of both doses if the initial dose is insufficient to achieve adequate reversal [4,8]. This packaging approach acknowledges the clinical reality that a single dose may be inadequate, particularly with potent synthetic opioids, and effectively represents a 4mg total dose for the approximately 2mg formulations. Our 4mg buccal dose (delivered as 2 x 2mg wafers, one in each cheek) directly mirrors this established clinical practice and total dose.

There are no significant concerns regarding the safety of the chosen doses as naloxone has been administered to healthy volunteers at considerably higher doses than those proposed in this study. Cohen et al. (1982) [31] administered naloxone at doses ranging from 0.3 to 4mg/kg intravenously to seven healthy volunteers, with the highest dose representing approximately 280mg for a 70kg individual. This study demonstrated dose-dependent increases in systolic blood pressure and respiratory rate but confirmed the overall safety and tolerability of high-dose naloxone in opioid-naïve individuals. Similarly, Volavka et al. (1979) [32] administered up to 20mg intravenously to healthy volunteers without serious adverse effects. These studies establish that our maximum proposed dose of 4mg buccally, which would be expected to result in lower systemic exposure than an equivalent intravenous dose due to incomplete absorption, falls well within established safety parameters.

4.2. Scientific Rationale

Buccal delivery is a potential alternative for emergency drug administration, and the route has been successfully used as a mode of delivery for emergency midazolam in the treatment of status epilepticus by non-medical persons. [6,33,34] Applying a tablet to the inner cheek is straightforward and can be performed by both non-specialist bystanders and healthcare professionals in emergency situations. After naloxone is released from the dosage form, the buccal mucosa, a stratified epithelium 40−50 cells (500−600 μm) thick, serves as the primary absorption barrier.

The vasculature of the buccal mucosa drains into the retromandibular, lingual, and facial veins, which then drain directly into the internal jugular vein and the systemic circulation via the superior vena cava [27,28,35]. While the absorption rate of naloxone from the human buccal cavity is not well known, studies in rats have shown a bioavailability of 70% from buccal administration compared to 0.3% via the oral route, owing to extensive first-pass metabolism, with maximum plasma levels reached within 15 minutes [29]. Additionally, sublingual administration of 2−8 mg of naloxone solution has been reported to induce opiate withdrawal in humans within 30 minutes [4,29].

Our feasibility work demonstrated that rapid-dispersal naloxone wafers can be produced, disintegrating within seconds [33]. Following this proof-of-concept laboratory production of a novel amorphous instant disintegrating tablet of naloxone, we have more recently shown feasibility and stability in a manufacturing facility according to good manufacturing practice standards (recent unpublished report available on request, manufactured by Catalent, Swindon, utilising their Zydis® technology). The formulation, comprising mannitol, gelatin, and sodium bicarbonate, are integral elements of the naloxone instant disintegrating buccal tablet [33].

Our innovative ultra-portable, dose-flexible, rapid-dispersal concentrated naloxone buccal wafer meets the challenge by bringing portability, speed, and dose-flexibility to the emergency overdose situation. Impact and benefits include an increased likelihood of naloxone being present and administered, and greater preparedness for unexpected high-dose or multiple-victim overdose scenarios. These will fit into a wallet or purse, thereby remedying current poor carriage rates for naloxone (only 15-20%).

The proposed buccal naloxone wafers have extreme portability: universal carriage is thus realistic. Current forms of naloxone need to be carried in their own container for the pre-filled syringe, or in a cardboard box or plastic case for nasal sprays. In contrast, buccal naloxone will be inserted into a wallet or purse in the credit-card section, thereby integrated with daily living and without needing repeated decision-making to achieve good adherence. The World Health Organization's objective is that ‘90% of all those with take-home naloxone must have it on them, or near them, at time of future emergency need’: but current naloxone carriage rates are only 10-20%. However, with ultra-portable buccal naloxone, the objective is realistic and potentially achievable. No current naloxone product even approaches this degree of portability.

Our recently completed Phase 1 feasibility testing met all five objectives. We demonstrated feasibility of production: stable lyophilised naloxone, dose range (1-4mg), dispersal (<3 seconds), multi-dose credit-card format, planned price below current formulations, and the declared objective is to steer the process forward to become a product with low cost and wide availability.

Likewise, phase 1 acceptability studies were strong, with 85% of one of the target populations of drug user peers willing to administer buccal naloxone (versus 78% nasal, 60% IM), and with

a willingness to carry buccal naloxone on their person at all times at 92% (versus 60% and 33% respectively).

In this new proposed healthy volunteer study, we will examine the blood levels achieved, and how rapidly, following administration of the novel buccal naloxone (at two doses) and sublingual (one dose) and we will compare these with plasma levels following administration of naloxone by current approved routes of IM (main reference route) and IN (secondary reference route). This will guide decision-making about any possible further attention needed to speed of absorption and/or overall absorption and half-life which will guide decisions about whether and how to proceed to future commercial product.

5. Trial Objectives and Design

5.1. Trial Objectives

Primary Objective

1. To characterize and compare the pharmacokinetic profile of a novel buccal formulation of naloxone in healthy volunteers with approved formulations (naloxone ampoule) as the injectable form, and Nyxoid® as naloxone nasal spray comparison.

Secondary Objectives

1. To measure the dose-proportionality of the novel formulation by comparing the plasma exposure of two different buccal doses and one sublingually.
2. To assess the safety and tolerability of the novel buccal formulation

5.2. Primary endpoints

The primary endpoint will be Time to Maximum Plasma Concentration (T_{max}).

The time to reach maximum plasma concentration will be determined to characterize the rate of naloxone absorption. T_{max} will be reported as the median and range for each treatment group, based on observations over the 4-hour post-dose period.

5.3. Secondary endpoints

The following secondary endpoints will be assessed to provide a comprehensive pharmacokinetic profile of the novel buccal and sublingual naloxone formulation in comparison to the IM and IN reference formulations:

Time to 50% of maximum concentration (T50%)

A particularly important secondary endpoint will be the time taken to reach 50% of the observed C_{max} as the increase in plasma levels before reaching C_{max} is unlikely to be linear and we consider the time taken to reach 50% with different formulations is likely to give an indication of the speed with which there may be future benefit of reversal of opioid overdose. T50% will be reported as the median and interquartile range for each treatment group, based on observations over the 4-hour post-dose period.

Maximum Plasma Concentration (C_{max})

The maximum observed plasma naloxone concentration will be determined for each formulation and dose. C_{max} will be reported as the mean and standard deviation for each treatment group, based on measurements taken over the 4-hour post-dose period.

Area Under the Curve from time zero to infinity (AUC_{inf})

The total systemic exposure to naloxone will be quantified by calculating the area under the plasma concentration-time curve extrapolated to infinity. AUC_{inf} will be reported as the mean and standard deviation for each treatment group, calculated from time zero to infinity.

Area Under the Curve from time zero to 15 minutes (AUC_{0-15})

Early systemic exposure to naloxone will be assessed by calculating the area under the plasma concentration-time curve from administration to 15 minutes post-dose. AUC_{0-15} will be reported as the mean and standard deviation for each treatment group.

Elimination Half-life ($t_{1/2}$)

The elimination half-life of naloxone will be calculated from the terminal slope of the log concentration-time curve. $t_{1/2}$ will be reported as the mean and standard deviation for each treatment group over the 4-hour post-dose period.

5.4. Trial Design

This is a Phase 1 CTIMP. This study will employ an open-label, within-subject design with repeated sessions to evaluate the IMPs in two stages. Stage 1 will use a five -way crossover design where each participant will receive all the considered treatment conditions in a random sequence.

The data from stage 1 will be used to select the IMPs for further evaluation in stage 2 according to the following criteria:

- Continue the evaluation of buccal formulation (2mg) when AUC_{0-15}min of this IMP is at least 90% of the AUC_{0-15}min of the approved Naloxone ampoule
- Continue the evaluation of buccal formulation (4mg) when AUC_{0-15}min of this IMP is at least 90% of the AUC_{0-15}min of the approved Naloxone ampoule
- Continue the evaluation of sublingual naloxone wafer (2mg) when AUC_{0-15}min of this IMP is at least 90% of the AUC_{0-15}min of the approved Naloxone ampoule

If none of these are satisfied, the study will stop at stage 1. When at least one of the IMPs is selected based on the stage 1 data, another cross-over design will be implemented on another group of participants.

A trial flowchart by visit time for stage 1 of the study is provided in section 5.5. The number of arms (and so the experimental visits) for stage 2 of the study is dependent on the result of stage 1. Participants that partake in stage 1 will not be eligible to participate in stage 2.

Assessments at each experimental visit as per the study schedule of assessments are provided in section 8.1.

Sample sizes are as detailed within section 11.2.

5.5. Trial Flowchart

Table 1 Clinical Trial Plan by Visit

Procedure		Experimental visits[1]					Follow-up
	Screening ≤ 28 days prior to IMP administration/Visit 1	Visit 1	Visit 2	Visit 3	Visit 4	Visit 5	7-14 days post-final visit (telephone/ online)
Written informed consent	X						
Demographic information[2]	X						
Medical assessment[3]	X						
Eligibility assessment/review	X	X	X	X	X	X	
Urine pregnancy test	X	X	X	X	X	X	X[8]
Urine drug screen	X	X	X	X	X	X	
Alcohol breath test	X	X	X	X	X	X	
IV cannulation		X	X	X	X	X	
Blood sampling (FBC, LFTS, U&Es)[4]	X						
Urinalysis[4]	X						
Vital signs[5]	X	X	X	X	X	X	
ECG	X						
Randomisation		X					
IMP administration[6]		X	X	X	X	X	
Administration site check[11]		X	X	X	X	X	
PK Sampling[7]		X	X	X	X	X	
Buccal Wafer Disintegration time [10]		X	X	X	X	X	
Visual Analogue Scale (VAS)		X	X	X	X	X	
AE Review[9]	←――――――――――――――――――――――――→						
Conmed Review[9]	←――――――――――――――――――――――――→						

Participants will be discharged on the same day as admission for each experimental visit.

1. Minimum 36 hrs between experimental visits. Breakdown of experimental visits in Section 8.1
2. Demographic information obtained will include sex, age, BMI, weight/height.
3. Includes review of pre-existing conditions, physical examination, medical, smoking, drug and alcohol history, and a conmed review of all medical products (incl. over-the-counter and herbal)
4. List in full in Section 8.3
5. Heart Rate (during experimental visit recorded via ECG), Respiratory Rate, Blood Pressure, Temperature, taken in supine position at specific timepoints in each experimental visit (pre-dose, 10 min, 30 min, 1 hr and 4hrs).
6. Five-way crossover design, in a randomised order for each participant on each study visit.
7. PK Sampling times detailed in Section 8.2.
8. WOCBP will be asked to confirm the result of a urine pregnancy test completed on that day, previously provided by the research team on the last dosing visit
9. All AEs and Conmed medications will be captured starting from the moment informed consent is obtained until the completion of follow-up. Adverse Event reviews to be conducted at screening, and specific timepoints during each experimental visit (pre-dose and 10 min, 30 min, 1 h and 4 h post IMP administration) and at follow-up (see Section 8.2)
10. Wafer disintegration time to be recorded for buccal/sublingual formulations only.
11. Administration site check at specific timepoints (pre-dose, 30 min and 4 hrs).

6. Trial Medication

6.1. Investigational Medicinal Product

The study will use three formulations of naloxone:

1. Novel rapid-dispersal buccal and sublingual naloxone wafer (IMP)
2. Intramuscular naloxone (IM naloxone), standard product
3. Intranasal spray naloxone (IN naloxone), standard product

Novel rapid-dispersal buccal and sublingual naloxone wafer:
The investigational product is a novel rapid-dispersal ultra-portable formulation of naloxone hydrochloride (HCl) in wafer form. It will be tested at two doses: 2mg and 4mg (2 x 2mg wafers) administered buccally, along with a 2mg dose administered sublingually. The excipients include mannitol, gelatine and purified H2O. The wafers will be manufactured and supplied by Catalent, utilising their established Zydis® technology platform.

Comparators: Naloxone as IM injection and IN spray
The IM formulation will be a naloxone ampoule which contains naloxone hydrochloride as a 0.4mg/1ml. The IN spray formulation will be Nyxoid®, containing 1.8mg/0.1mL naloxone hydrochloride dihydrate, equivalent to 2 mg of naloxone hydrochloride, per dose.

The 0.4mg IM dose is widely recognized as the standard initial dose for opioid overdose reversal in clinical practice. [1,36]Multiple studies have demonstrated the safety and efficacy of this dose in both clinical and healthy volunteer settings. [37,38] Using this standard dose allows for a direct comparison with real-world clinical use, enhancing the translational value of our study.

The 1.8mg/0.1mL IN naloxone dose (Nyxoid®) was selected based on the following rationale:
a) This is the approved dose for the Nyxoid® nasal spray in Europe and other regions. [39] b) Studies have shown that this dose provides plasma concentrations over the first 10 minutes comparable to the 0.4mg IM dose, which is crucial for our comparative pharmacokinetic analysis. [26,40] c) Higher doses of naloxone have been widely used clinically in the US with

the 4mg spray (Narcan) being the most widely used product, and even higher doses of IN naloxone (up to 8mg) have been approved in the US for clinical use, and 8 mg nasal spray Kloxxado. [38,41,42]

For the naloxone wafer, we anticipate bioavailability comparable to the nasal spray and the maximum dosage being administered in the healthy volunteers' study is at half the level contained within the recently approved 8 mg naloxone nasal spray in the US.

6.1.1. Packaging and Labelling:

The information presented on the IMP labels will be annex 13 compliant. The buccal and sublingual wafers will be packaged in individual, sealed, moisture-resistant blisters. Each blister will be packaged in a labelled sealed carton which will be labelled with the study number, batch number, dose strength, unit quantity, expiry date, storage and administration instructions (store at 15–25 °C; protect from light/moisture; do not freeze; shelf-life per IB/IMPD) according to annex 13 requirements. Manufacture, primary packaging and testing will be conducted at Catalent UK Swindon Zydis Ltd; secondary packaging and clinical-trial labelling at Catalent CTS (Edinburgh) Ltd, Bathgate facility. Once shipped and subsequently received by South London and Maudsley NHS Foundation Trust (SLaM) Pharmacy, additional study/subject-specific (dispensing) labels will be added to the cartons to clarify usage within specific different treatment arms of the naloxone wafer (eg, two cartons to be dispensed for administration for the 2x2mg (4mg) visit).

IM naloxone ampoule and IN naloxone nasal spray (Nyxoid®) will be supplied according to the pharmacy manual. Annex 13 compliant labels will also be used for these comparators and provided by SLaM Pharmacy.

SLaM Pharmacy will dispense IMP to the King's Clinical Research Facility (CRF) where the trial will take place.

6.1.2. Regulatory status of IMP

For the comparators, IM and IN naloxone, as licensed medications, the Summary of Product Characteristics (SmPC) for each will be used for the clinical management of the drug and product characteristics, including any expected adverse events.

Table 2 Marketing authorisation numbers of IMP

Formulation	Route	Dose strength	MA number
Naloxone ampoule	IM injection	0.4mg (0.4mg/1ml)	PL 01502/0141<br>PL 03551/0109
Nyxoid® nasal spray	IN spray	1.8mg (1.8mg/0.1ml)	PLGB 16950/0374

As a non-licensed experimental IMP, the novel rapid-dispersal naloxone wafer does not have marketing authorisation in the UK. Product characteristics can be found in the Investigator Brochure (IB).

Quality, manufacture and control details are provided in the IMPD (Drug Product, Catalent UK Swindon Zydis Ltd; Final v1.0, 04-Jul-2025) and the supporting IMPD (Drug Substance, MacFarlan Smith Ltd; Final v1.0, 04-Jul-2025).

6.2. Dosing Regimen

In stage 1, this study will employ a randomised, five-way crossover design to compare the novel naloxone formulation with existing IM and IN formulations. Each participant will receive all five treatments in a randomised order, with a washout period of at least 36 hours between each dosing session (thus permitting the next session two days later).

Route of Administration and Dosage:

1. Buccal Naloxone:
   - 2mg (1 x 2mg wafer)
   - 4mg (2 x 2mg wafers)

Participants will be positioned in the supine position for administration. The wafer(s) will be placed between the lower gum and cheek, allowing it to dissolve. Following placement, gentle external massage of the cheek area will be applied for 30 seconds to promote adherence of the wafer to the mucosa and enhance drug absorption. For the 4mg dose, a single 2mg wafer will be administered into each cheek of the participant, with the second wafer administered immediately following the first in the other cheek, to total a 4mg administration. Buccal wafer administration time will begin from the administration of the second wafer.

Drug administration will be performed by appropriately trained and delegated research staff who have received standardised training on the specific administration procedures for each formulation. A medically qualified doctor will be immediately available on-site during dosing and the early PK window, with resuscitation equipment available. The dose administrator can be non-medical, e.g. a trained lay-person to closely follow the intended posology. Within this clinical trial, administration responsibilities are delineated as follows: intramuscular dosing will be conducted by appropriately trained, delegated clinical personnel; intranasal, buccal, and sublingual administration will be performed by trained, delegated clinical research facility staff. The reference to lay-person administration serves solely as methodological rationale to reflect anticipated real-world utilization patterns.

2. Sublingual Naloxone
   - 2 mg wafer

Administered by placing the wafer under the tongue, allowing it to dissolve.

3. IM Naloxone:
   - 0.4 mg/1ml ampoule

Administered via IM injection in the deltoid muscle.

4. IN (Nyxoid®) Spray Naloxone:
   - 1.8 mg/0.1ml

Administered as a single spray into one nostril.

The order of administration will be randomised for each participant as per section 7.5.

6.2.1. Duration of Participant Participation:

The minimum washout period between drug administrations will be 36 hours (naloxone's half-life is <2 hours). A participant could therefore complete all study visits in stage 1, including follow-up, within three weeks.

The maximum period of each participant's participation is expected to be less than three months. If a participant has not completed all dosing sessions within this period, the screening visit will be repeated to ensure that they continue to meet all inclusion/exclusion criteria.

6.3. IMP Risks

The risks associated with this study are expected to be minimal. Naloxone is a well-established opioid antagonist with a good safety profile, with few adverse effects. However, as this investigational medicinal product is a novel wafer formulation of naloxone, no formulation-specific safety profile has yet been established.

The Reference Safety Information for the study will be Section 7 of the Investigators' Brochure. Information for products with marketing authorisation (e.g. comparators) can be found within the summary of product characteristics (SmPC) for that product.

For ease of reference, the known adverse reactions for naloxone (based on post-marketing experience and the current SmPC) are summarised in Table 3 below.

Table 3. Adverse reactions to marketed naloxone

System Organ Class	Frequency	Adverse Reactions
Immune system disorders	Very rare	Allergic reactions (urticaria, rhinitis, dyspnoea, Quincke's oedema), anaphylactic shock
Nervous system disorders	Common	Dizziness, headache
	Uncommon	Tremor, sweating
	Rare	Seizures, tension
Cardiac disorders	Common	Tachycardia
	Uncommon	Arrhythmia, bradycardia
	Very rare	Cardiac fibrillation, cardiac arrest
Vascular disorders	Common	Hypotension, hypertension
Respiratory, thoracic and mediastinal disorders	Uncommon	Hyperventilation
	Very rare	Pulmonary oedema
Gastrointestinal disorders	Very common	Nausea
	Common	Vomiting
	Uncommon	Diarrhoea, dry mouth
Skin and subcutaneous tissue disorders	Very rare	Erythema multiforme
General disorders and administration site conditions	Common	Postoperative pain
	Uncommon	Irritation of vessel wall (after IV administration); local irritation

		and inflammation (after IM administration)
The following frequency terminology is used:<br>Very common: ≥ 1/10;<br>Common: ≥ 1/100, < 1/10;<br>Uncommon: ≥ 1/1,000, < 1/100;<br>Rare: ≥ 1/10,000, < 1/1,000;<br>Very rare: < 1/10,000;<br>Not known (cannot be estimated from the available data)		

6.3.1. Pharmacokinetic interactions

Due to the exclusion criteria of this study, the risk of drug interactions is minimized. Participants taking any type of medication, whether prescription or over-the-counter (with the exception of contraceptives), will not be allowed to participate in the study. This includes any form of opioid medication. The exclusion criteria also exclude anyone using opioids non-medically/recreationally. As a result, the potential for drug interactions with naloxone is minimal. However, participants will still be instructed to avoid any new medications, herbal supplements, or recreational substances for the duration of the study to maintain the integrity of the pharmacokinetic data. Naloxone is metabolised by UGT enzymes and is not known to inhibit or induce any CYP enzymes. We therefore do not expect that there is any risk of an interaction with contraceptive medications.

Risk mitigation strategies:

- Exclusion criteria: Individuals with known hypersensitivity to naloxone or any components of the formulations will be excluded from the study. Those with recent use of opioids or prohibited medications will also be excluded.
- Medical screening: All participants will undergo thorough medical screening to ensure they are healthy volunteers without pre-existing conditions that could increase risk.
- Medication review: A comprehensive review of all medications, including over-the-counter and herbal products, will be conducted before enrolment and before each dosing session.
- Participant education: Participants will be thoroughly educated about prohibited substances and medications and the importance of adherence to these restrictions.
- Monitoring: Participants will be closely monitored for adverse events, particularly during and immediately after drug administration.
- Emergency preparedness: Although not expected to be needed, emergency medical equipment and trained personnel will be available during all study procedures.

6.4. Drug Accountability

IMP accountability will be maintained throughout the study. If IMP is dispensed by the Maudsley Pharmacy but not administered (for example, if a participant withdraws during an experimental visit), it must be returned to the Maudsley Pharmacy. Returned IMP will be quarantined and not discarded until the CRA has verified the accountability records. Destruction will take place only after Sponsor authorisation and will be performed in accordance with local SOPs with documentation retained.

6.5. Storage of IMP

SLaM Pharmacy will dispense the IMP and maintain accountability. At study completion, unused study drug will be destroyed per local procedures. Storage conditions are as defined in the IB, and where applicable, the SmPC.

6.6. Participant Compliance

Participants will not be issued take-home IMP; all dosing occurs on-site under direct supervision by delegated staff. Accordingly, participant compliance is not applicable. Visit adherence will be monitored via attendance and completion of on-site procedures. For IMP handling, returns and destruction, see 6.4 Drug Accountability.

6.7. Concomitant Medication

Apart from contraceptives, concomitant medications are not permitted during the study and are an exclusion criterion.

While naloxone itself is not known to have direct teratogenic effects, the administration of an opioid antagonist and the associated physiological responses could potentially induce stress that may not be ideal for foetal development. Additionally, as a precautionary measure in early-phase clinical trials, it is crucial to minimize any unforeseen risks to a developing foetus. Therefore, female participants of child-bearing potential* should use an effective method of contraception** for the duration of the trial and will be advised to continue this for 2 weeks after the completion of their participation in the trial.

Over-the-counter medications for minor ailments (e.g., acetaminophen for headaches) may be permitted on a case-by-case basis but will be checked on by the investigator and their team at each visit. These should be avoided for 48 hours before each dosing session unless absolutely necessary.

Rescue medication may be administered at the Investigator's discretion for the management of anticipated symptoms (e.g., paracetamol for headache or pyrexia, oral/IV fluids for dehydration, antiemetics such as ondansetron for nausea/vomiting). All rescue medication will be recorded as concomitant medication in the CRF.

*A woman is considered of childbearing potential (WOCBP), i.e. fertile, following menarche and until becoming post-menopausal unless permanently sterile. Permanent sterilisation methods include hysterectomy, bilateral salpingectomy, and bilateral oophorectomy. A postmenopausal state is defined as no menses for 12 months without an alternative medical cause. A high follicle-stimulating hormone (FSH) level in the postmenopausal range may be used to confirm a postmenopausal state in women not using hormonal contraception or hormonal replacement therapy. However, in the absence of 12 months of amenorrhea, a single FSH measurement is insufficient.

** Methods that can achieve a failure rate of less than 1% per year when used consistently and correctly are considered as highly effective birth control methods. Such methods include:

- combined (oestrogen and progestogen-containing) hormonal contraception associated with inhibition of ovulation:
    - oral

    - intravaginal
    - transdermal
- progestogen-only hormonal contraception associated with inhibition of ovulation:
    - oral
    - injectable
    - implantable
- intrauterine device (IUD)
- intrauterine hormone-releasing system (IUS)
- bilateral tubal occlusion
- vasectomised partner
- sexual abstinence (abstinence should only be used as a contraceptive method if it is in line with the subjects' usual and preferred lifestyle. Periodic abstinence (calendar, symptothermal, post-ovulation methods) is not an acceptable method of contraception)

7. Selection and Withdrawal of Participants

7.1. Inclusion Criteria

I.01. Healthy volunteers. Defined as healthy based on medical examination which includes: clinical history, physical examination, ECG, vital signs, and laboratory tests of blood and urine.

I.02. Age 18-60

I.03. Able and willing to provide written informed consent

I.04. Adequate venous access and willingness for intravenous cannulation during each visit.

7.2. Exclusion Criteria

E.01. Clinically relevant medical history, physical findings, ECG, or laboratory values at the pre-trial screening assessment that could interfere with the objectives of the trial or the safety of the participant.

E.02. Presence of acute or chronic illness or history of chronic illness sufficient to invalidate the volunteer's participation in the trial or make it unnecessarily hazardous.

E.03. Impaired endocrine, thyroid, hepatic, respiratory or renal function, diabetes mellitus, coronary heart disease, or history of any neurological or mental illness.

E.04. Surgery or medical condition that might affect the absorption of medicines.

E.05. Blood pressure and heart rate in the supine position at the screening examination outside the ranges: blood pressure 90–140 mm Hg systolic, 40–90 mm Hg diastolic; heart rate 40–100 beats/min. Repeat measurements are permitted if values are borderline (i.e. values that are within 5 mm Hg for blood pressure or 5 beats/min for heart rate) or if requested by the investigator. Subjects can be included if the repeat value is within range or still borderline but deemed not clinically significant by the investigator.

E.06. Loss of more than 400 mL of blood during the 3 months before the trial, e.g. as a blood donor.

E.07. Any prescribed medication (apart from contraceptives).

E.08. Use of any over-the-counter medications containing codeine or other opioids, prescribed opioid medication, or illicitly obtained opioids within the past 2 weeks (if the

participant is taking a long-acting opioid the period might, after consideration by the examining doctor, be extended to 4 weeks or longer according to the washout period).

E.09. BMI <18 or >30.0kg/m2.

E.10. Intake of more than 14 units of alcohol weekly.

E.11. Pregnant or breastfeeding.

E.12. Women of childbearing potential (as defined in CTFG guidelines, see 6.7 Concomitant Medication) not willing to use a highly effective form of contraception (as defined in CTFG guidelines, see section 6.7 Concomitant Medication) during participation in the study or male patients not willing to ensure the use of a condom during participation in the study.

E.13. eGFR≤ 70 ml/min.

E.14. Any liver function or renal function test abnormality. A repeat is allowed on one occasion for determination of eligibility.

E.15. Urine drug screen positive for any substances.

E.16. Positive alcohol breath test, above 0.

E.17. Participant in any other clinical trial or experimental drug study in the past 3 months

E.18. Known hypersensitivity to naloxone and/or formulation excipients (gelatin, mannitol).

E.19. Not willing to ingest fish-derived gelatin.

E.20. Insufficient understanding of the trial.

7.3. Selection of Participants

7.3.1. Recruitment

Participants will be identified by word of mouth, university recruitment emails, and paper and online advertisements. All adverts/recruitment materials will be REC-approved prior to use. Participants may contact the research team via email in response to the recruitment advertisements if they are interested in taking part in the study.

7.3.2. Pre-screening

Prior to screening, the participant will be emailed a Participant Information Sheet (PIS). This will be done at least 24 hours before the screening visit. Potential participants will be allowed as much time as needed to decide whether or not to participate. They will then be contacted by one of the researchers by telephone or video call to be screened based on the inclusion and exclusion criteria and to gather contact information. They will have the opportunity to ask the researcher questions about the study during the call, be informed of their right to withdraw, and be asked if they have read and understood the PIS.

If the participants meet the initial inclusion and exclusion criteria, they will be invited for a screening visit.

7.4. Consent

Informed consent will be obtained by a physician prior to the participant undergoing procedures that are specifically for the purposes of the trial. The right of a participant to refuse participation without giving reasons will be respected. The participant will remain free to withdraw at any time from the trial without giving reasons. They will be provided with a contact point where they may obtain further information about the trial. Potential participants should

be able to give consent, and a person is assumed to have the mental capacity to decide unless it is shown to be absent. Consent will be taken by medical doctors who are duly authorised, trained and competent to participate according to the ethically approved protocol, principles of Good Clinical Practice (GCP) and Declaration of Helsinki.

Participants will be informed that blood samples collected during the study will be used solely for the purpose of this study, pharmacokinetic analysis to measure naloxone levels. Only plasma will be stored for analysis and no human tissue will be retained at the end of the research. All biological samples will be handled, transported, stored, accessed, and processed in accordance with the 2004 Human Tissue Act. Similarly, all samples will be disposed in accordance with the Human Tissue Authority's Code of Practice.

Written material consisting of participant information leaflet and consent documentation will be approved by the Research Ethics Committee (REC) and will comply with GCP, local regulatory and legal requirements. There will be the opportunity for the participant to ask questions to a member of the research team. The patient will be given as much time as required to consider the information and consider their participation. A copy of the consent form and PIS will be given to the participant, with the original consent filed in the investigator site file and a copy of the consent form in the Case Report Form.

7.5. Randomisation Procedure / Code Break

This study will employ an open-label, within-subject design with repeated sessions. Each participant in stage 1 of the study will receive all five treatment conditions in a random sequence. A randomisation schedule will be created using five treatment conditions with a corresponding code as per Table 2.

Table 3 Treatment Coding for Randomisation

Treatment Code:	Drug administration and dosage	IMP/Comparator
A	Buccal administration of 2mg naloxone rapid dispersal wafer	IMP
B	Buccal administration of 4mg naloxone (2x 2mg) rapid dispersal wafers	IMP
C	Sublingual administration of 2mg naloxone rapid dispersal wafer	IMP
D	IM administration of 0.4mg/1ml naloxone ampoule	Comparator
E	IN administration of 1.8m/0.1ml naloxone (Nyxoid®)	Comparator

Another randomisation schedule will be created accordingly for stage 2 of the study if the study does not terminate early (i.e., when at least one of the IMPs is selected for further evaluation)

7.5.1. Method of Assignment to Treatment Ordering

Each participant will be assigned a unique study number upon pre-screening (P001; P002, P003 etc), screening (e.g. S001, S002, S003 etc), and randomisation (R001, R002, R003 etc), allocated sequentially in the order of assignment.

The unique randomisation study number allocated to each participant will correspond with the randomisation schedule. The randomisation schedule will determine the order of treatment for each participant.

The randomisation schedule will be stored securely and will only be accessible to authorized personnel not directly involved in participant assessments.

7.5.2. Randomisation

At each experimental visit, the appropriate treatment will be administered according to the pre-determined randomisation schedule.

If a participant withdraws from the study before completing all dosing visits, a new randomisation number will be assigned to any replacement participant, no randomisation number will be re-used.

Randomisation service will be provided by the King's Clinical Trial Unit (CTU).

Each randomised participant will be provided with a card detailing emergency contact details. Participants will be requested to carry this card with them at all times while participating in the trial.

7.5.3. Emergency Code Break

As this is an open-label study, there is no need for a code break procedure. The treatment allocation for each session will be known to both the investigators and participants.

7.6. Withdrawal of Participants

i. Withdrawal of consent.
ii. Significant abnormalities detected during the medical assessment or blood tests at the screening visit.
iii. Suspected Unexpected Serious Adverse Reaction.
iv. Participant does not attend follow-up point.
v. Investigator discretion.

Session–specific exclusions:
If a participant meets any of the following session-specific exclusion criteria, the session will be postponed and rescheduled for a later date. The participant will remain enrolled in the study and can complete the remaining sessions as planned, provided they meet all criteria at the time of the rescheduled session.

i. Use of any over-the-counter medications containing codeine or other opioids, prescribed opioid medication or of illicitly obtained opioids within the past 2 weeks (if the participant is taking a long-acting opioid the period might, after consideration by the examining doctor, be extended to 4 weeks or longer according to washout period).
ii. Urine drug screen positive for any substance on the day of the session.
iii. Positive alcohol breath test (above 0) on the day of the session.
iv. Presence of any acute respiratory symptoms (such as cough, shortness of breath, sore throat), suspected/confirmed COVID-19, other respiratory infection or any other acute disease at the investigator's discretion.

Participants have the right to withdraw from the study at any time for any reason. The investigator also has the right to withdraw participants from the study drug in the event of inter-current illness, AEs, SAEs, SUSARs, protocol violations, administrative reasons, or other reasons. It is understood by all concerned that an excessive rate of withdrawals can render

the study un-interpretable; therefore, unnecessary withdrawal of participants should be avoided. Should a participant decide to withdraw from the study, all efforts will be made to report the reason for withdrawal as thoroughly as possible.

If participants are withdrawn prior to completing all five experimental visits and are therefore missing a full dataset from experimental visits, they will be replaced. Participants lost to follow-up will not be replaced.

In the event of early withdrawal, any data already obtained, such as pharmacokinetic and safety, can be retained by the study and may be used in analyses and presented in publications.

Withdrawal Due to Missed Visits:
Since this is a 5-way crossover requiring all treatments for analysis, missing 2 consecutive scheduled visits or 3 total visits should constitute withdrawal, as incomplete data would compromise the crossover design.

Lost to follow up:
For contact attempts before classifying participants as lost to follow-up we would make three contact attempts over a one-week period using multiple communication methods including phone calls, emails, and text messages if participants have consented to receive them. These attempts should be spaced at 24 hours, three days, and seven days after a missed appointment, with each attempt using a different contact method when possible. All contact attempts should be thoroughly documented in the source documents, and participants should be classified as lost to follow-up only after all three attempts have failed to elicit any response.

7.7. Expected Duration of Trial

The end of the trial will be defined as database lock.

8. Trial Procedures

The overall trial design for each participant, from screening to final follow-up is detailed in 5.5 Trial Flowchart. The study schedule of assessments (section 8.1) details timing of procedures planned for experimental visits 1-5, pre-dose and post-dose.

As per 6.2 Dosing Regime, there will be a minimum of 36 hours between each of the five experimental visits.

A breakdown of all study assessments is detailed by visit in section 8.2.

8.1. Study Schedule of Assessments

Table 4 Assessment Timepoints for each of the Experimental Visits in Stage 1

Assessment	Timepoint																
	Pre-dose		Post-dose														
	Pre	-5m	0m	2m	4m	6m	8m	10m	12.5m	15m	30m	45m	1h	1.5h	2h	4h	> 4h
Eligibility assessment/review	X																
Urine pregnancy	X																
Urine drug screen	X																
Alcohol breath test	X																
IV cannulation	X																
Vital sign assessments[1]	X							X			X		X			X	
Treatment allocation/ randomisation[2]	X																
IMP administration[3]			X														
Administration site check[4]	X										X					X	
PK blood sampling		X		X	X	X	X	X	X	X	X	X	X	X	X	X	
Wafer disintegration time[5]			X														
Visual Analogue Scale (VAS)[6]			X														
Discharge																	X[7]
AE Review[8]		X						X			X		X			X	
Conmed review	←→																

In this five-way crossover design study in stage 1, all treatment experimental visits will follow this schedule.

1. Vital sign assessments include. Heart rate, respiratory rate, blood pressure, and oral body temperature.
2. Each participant will be assigned a randomised treatment allocation sequence for drug administration ordering either at or before visit 1, pre-dose.
3. IMP administration is allocated according to the randomised treatment allocation ordering, determined at visit 1, pre-dose.
4. For buccal and sublingual treatment arms, oral mucosa will be examined with any changes documented. IM injection site and nasal cavities will be examined in the other arms.
5. Wafer disintegration time only recorded for buccal and sublingual naloxone administration visits.
6. VAS assessment completed by participants immediately following administration of each oral formulation to assess taste and tolerability.
7. Discharge at least 4 hours post dose at minimum.
8. AE review reported at anytime, however participants will be prompted by research staff at these timepoints.

8.2. By Visit

All in-house visits will be performed and held at the CRF. Trial procedures will be performed by a trained member of the research team or CRF staff.

8.2.1. Screening Visit

The screening visit will be performed at a maximum of 28 days prior to IMP administration/Visit 1. At the end of the visit, each participant will be provided with a card detailing emergency contact details.

The following interventions and assessments will be completed in order:

i. Informed consent
ii. Collection of demographic information
iii. Medical review and physical examination (including review and documentation of current medications).
iv. Height, weight, BMI
v. Review of eligibility
vi. Urine sample for urinalysis, pregnancy, and illicit drug testing (see section 8.3 for full list)
vii. Alcohol breath test
viii. Blood tests: LFTs; FBC & U&Es (see section 8.3 for full list)
ix. Vital Signs
x. ECG

8.2.2. Visits 1-5: Experimental visits for Stage 1

1. Randomisation to the treatment schedule ordering at or before Visit 1 as per Section 7.5 and Section 11.3.
2. Administration of IMP:

   As this is an open-label study, participants will be informed of the dosing schedule. There will be a minimum 36 hrs washout between experimental visits.

   For all five visits, the administration site will be checked pre-dose, and 30min & 4h post.

   For buccal and sublingual IMP formulations, wafer disintegration time will be measured.
3. VAS:

   Participants will complete a 100mm VAS immediately after administration of each formulation to assess taste and local tolerability.
4. Blood sampling:

   Given the special interest in early absorption, blood collection included intense sampling during the first 15 minutes, with a total of 14 samples per session (pre-dose, at 2, 4, 6, 8, 10, 12.5, 15, 30 and 45 minutes and 1, 1.5, 2, 4 hours post-dose).
5. Vital signs:

   Vital signs (HR, RR, BP, Temp) will be recorded on experimental visits at the following times: pre-dose, 10 minutes, 30 minutes,1 hour, and 4 hours.
6. Adverse events and conmed checks:

   Participants will be asked to report adverse events at specific timepoints (pre-dose, 10 minutes, 30 minutes,1 hour, and 4 hours) during each experimental visit. Additionally, participants will be instructed that they can report any adverse events to the research

team at any time throughout their participation in the study. Conmed checks will be checked along with AEs.

The following interventions and assessments will be completed in order:

i. Eligibility review
ii. Review of any new medications or changes in medication since last visit
iii. Urine sample for pregnancy and illicit drug testing
iv. Alcohol breath test
v. IV cannulation
vi. Treatment allocation
vii. Pre-dose; 0-5mins: blood sampling; vital signs; adverse events assessment; administration site check
viii. 0 mins: drug administration; wafer disintegration time (for buccal and sublingual visits only); visual analogue scale
ix. 2 mins: blood sampling
x. 4 mins: blood sampling
xi. 6 mins: blood sampling
xii. 8 mins: blood sampling
xiii. 10 mins: blood sampling; vital signs; adverse events assessment
xiv. 12.5 mins: blood sampling
xv. 15 mins: blood sampling
xvi. 30 mins: blood sampling; vital signs; adverse events assessment; administration site check
xvii. 45 mins: blood sampling
xviii. 1 hour: blood sampling; vital signs; adverse events assessment
xix. 1.5 hrs: blood sampling
xx. 2 hrs: blood sampling
xxi. 4 hrs: blood sampling; vital signs; adverse events assessment; administration site check
xxii. > 4 hrs: discharge

8.2.3. Washout:

There will be a minimum 36 hrs washout between experimental visits.

8.2.4. Visit 6: Follow-up visit

The follow-up visit will be completed via remote video call (or phone call if video incapability) 7-14 days after the final dosing visit. Participants will be asked to report any adverse events. Female participants of childbearing potential will be asked to confirm the result of a urine pregnancy test completed on that day, previously provided by the research team on the last dosing visit.

8.2.5. Experimental visits for Stage 2:

The number of experimental visits in Stage 2 will depend on which treatment arms continue for further evaluation, based on the results of Stage 1. A minimum of 36 hours will be maintained between visits. The specific details of Stage 2 experimental visits will be determined after analysis of Stage 1 data and according to the criteria outlined in Section 5.4.

8.3. Laboratory Tests

At the screening visit, a blood sample will be taken from each participant to test for haematology (FBC) renal function (urea and electrolytes) and hepatic function (liver function tests). A urine sample will also be taken for Urinalysis. These tests will be completed by the study team and be processed according to standard local procedures.

Table 5 Laboratory Tests

**Full Blood Count (FBC)**		**Liver Function Tests (LFTs)**	**Urea & Electrolytes (U&Es)**	**Urinalysis**
White Blood Cell count	Lymphocyte Count	Total Protein	Sodium	Protein
Red Blood Cell count	Monocyte Count	Total Bilirubin,	Potassium	Blood
Haemoglobin	Eosinophil Count	ALP	Creatinine	Leucocytes
Haematocrit	Basophils	AST	Urea	Nitrate
Mean Cell Volume	Immature Granulocytes Count	GGT		Glucose
Mean Cell Concentration	Metamyelocytes Count	Albumin		Ketone
Red Cell Distribution Width	Myelocytes Count	Globulin		pH
Mean Cell Haemoglobin Concentration	Promyelocytes Count			Specific gravity
Platelet Count	Blasts Count			Bilirubin
Mean Platelet Volume	Nucleated RBC's			Urobilinogen
Neutrophil Count				

Urine samples for urinalysis, drug and pregnancy testing will be collected in standard collection pots. The samples will be disposed of as soon as the tests are successfully completed.

On day one of each experimental visit, participants will have a venous cannula inserted for the collection of blood samples. Blood samples will be collected in 5ml EDTA tubes.

Total blood volume collected per participant will not exceed 100ml for the study.

Within 30 min of collection, the samples will be centrifuged (3000rpm for 10min). The plasma will be decanted from the tube into two screw-cap collection tubes and immediately placed in a -20°C freezer in the CRF. The stored samples will be pseudo-anonymised with participant ID, visit number and time point. Only the research team will be able to link the ID number to the participant's details. At the end of each day, the sample will be moved to a -80°C freezer for longer-term storage in the King's CRF.

Only plasma will be stored so that analyses can be completed. No human tissue will be stored at the end of the research. Biological samples collected from participants as part of this trial will be transported, stored, accessed, and processed in accordance with the 2004 Human Tissue Act.

PK blood samples will be analysed at a KCL laboratory, Department of Nutritional Sciences. All other samples will be analysed either at the CRF or Synnovis, Kings College Hospital.

9. Assessment of Efficacy

9.1. Efficacy Parameters

9.1.1. Primary Efficacy Parameters

The primary efficacy parameter for this study is t_{max}.

9.1.2. Secondary Efficacy Parameters

a) Pharmacokinetic Parameters:

- Time to 50% of maximum concentration (T50%)
- Maximum Plasma Concentration (Cmax)
- Elimination Half-life (T1/2)
- Area Under the Curve (AUC) from:
  - Time zero to 15 minutes (AUC_{0-15})
  - Time zero to infinity (AUC_{inf})

b) Local Tolerability:

- Buccal and Sublingual Wafer Disintegration
- Visual Analogue Scale (VAS) for Taste and Tolerability

9.2. Procedures for Assessing Efficacy Parameters

The following procedures will be used to assess the efficacy parameters:

Blood Sampling:

- Venous blood samples (1 mL) will be collected via IV cannulation at the following time points for each formulation: 0 (pre-dose), 2, 4, 6, 8, 10, 12.5, 15, 30 and 45 minutes and 1, 1.5, 2, 4 hours after dosing. Each sample will be split into two plasma cryovials.
- Samples will be centrifuged within 30 minutes of collection, and plasma will be separated and stored at -20°C until the end of the visit, and then stored at -80°C until analysis.

Plasma Naloxone Analysis:

- Plasma concentrations of naloxone will be determined using a validated liquid chromatography-tandem mass spectrometry (LC-MS/MS) method.
- The following parameter will be recorded in the eCRF for each time point: plasma naloxone concentration (ng/mL).

Pharmacokinetic Analysis:

- AUC will be calculated using the linear trapezoidal method and extrapolated to infinity.
- C_{max} and T_{max} will be determined directly from the observed concentration-time data.
- t1/2 will be calculated using the terminal elimination rate constant.
- Relative bioavailability will be calculated by comparing the dose-normalized AUC0-∞ of buccal and sublingual naloxone to that of IM and IN formulations.

Buccal and Sublingual Wafer Disintegration:

- The time for complete disintegration of the buccal naloxone wafer will be measured and recorded by a study team member.
- Participants will be asked to report the moment they feel the wafer has completely disintegrated.
- The following will be recorded in the CRF: time to complete disintegration (seconds).

Visual Analogue Scale (VAS) for Taste and Tolerability:

- Participants will complete a 100mm VAS immediately after administration of each formulation to assess taste and local tolerability.
- The following will be recorded in the CRF: VAS score for taste (0-100), VAS score for tolerability (0-100).

All efficacy assessments will be conducted for each of the three formulations (buccal/sublingual, IM, and IN) and for each dose of the buccal formulation (2mg and 4mg). The data collected will be used to construct individual plasma concentration-time profiles, calculate pharmacokinetic parameters, and compare the efficacy of the buccal and sublingual naloxone formulation to the existing IM and IN formulations.

These procedures will provide comprehensive data to assess the efficacy of the novel buccal and sublingual naloxone formulation in terms of its pharmacokinetic profile, bioavailability, and user acceptability compared to existing naloxone formulations.

10. Assessment of Safety

10.1. Specification, Timing and Recording of Safety Parameters

The safety of participants will be monitored throughout the study using a combination of physical examinations, laboratory tests, and adverse event reporting. These safety assessments are designed to detect any potential adverse effects and to ensure participant well-being throughout the study.

The trial will take place at the CRF at King's College Hospital. This facility is equipped with emergency resuscitation services, ensuring enhanced safety for participants in case of any adverse events or emergencies.

Laboratory Tests: The following laboratory tests will be performed at screening:

- Full Blood Count (FBC): to assess overall health and detect any haematological abnormalities
    1. White Blood Cell count
    2. Red Blood Cell count
    3. Haemoglobin
    4. Haematocrit
    5. Mean Cell Volume
    6. Mean Cell Concentration
    7. Red Cell Distribution Width
    8. Mean Cell Haemoglobin Concentration
    9. Platelet Count
    10. Mean Platelet Volume
    11. Neutrophil Count
    12. Lymphocyte Count
    13. Monocyte Count
    14. Eosinophil Count
    15. Basophils
    16. Immature Granulocytes Count
    17. Metamyelocytes Count
    18. Myelocytes Count
    19. Promyelocytes Count
    20. Blasts Count
    21. Nucleated RBC's

- ➢ Liver Function Tests (LFTs): total protein, total bilirubin, AST, ALT, GGT, albumin and globulin to monitor for any hepatotoxicity
- ➢ Renal Function Tests (U&Es): Urea, creatine and Electrolytes (sodium and potassium) to assess kidney function
- ➢ Urinalysis: protein, blood, leucocytes, nitrate, glucose, ketone, pH, specific gravity, bilirubin and urobilinogen to screen for urinary tract infections or other abnormalities

Additional tests will be performed at the following time points:

- ➢ Pregnancy test for female participants: at screening, before each dosing session, and at the final follow-up visit
- ➢ Urine drug screen: at screening and before each dosing session

Special Considerations:

- ➢ As buccal administration may cause local irritation, the oral mucosa will be examined before and at four hours after each dose administration, with any changes documented via the use of photography if necessary. IM injection site and nasal cavities will be examined in the other trial arms.

10.2. Procedures for Recording and Reporting Adverse Events

The Medicines for Human Use (Clinical Trials) Regulations 2004 and Amended Regulations 2006 gives the following definitions:

- **Adverse Event (AE)**: Any untoward medical occurrence in a participant to whom a medicinal product has been administered, including occurrences which are not necessarily caused by or related to that product.
- **Adverse Reaction (AR):** Any untoward and unintended response in a participant to an investigational medicinal product which is related to any dose administered to that participant.
- **Unexpected Adverse Reaction (UAR)**: An adverse reaction the nature and severity of which is not consistent with the information about the medicinal product in question set out in the reference safety information from:

  The summary of product characteristics (SmPC) for that product (for products with a marketing authorisation)

  The Investigator's Brochure (IB) relating to the trial in question (for any other investigational product)

- **Serious adverse Event (SAE), Serious Adverse Reaction (SAR) or Suspected Unexpected Serious Adverse Reaction (SUSAR):** Any adverse event, adverse reaction or unexpected adverse reaction, respectively, that**:**
  - o results in death;
  - o is life-threatening;
  - o required hospitalisation or prolongation of existing hospitalisation;
  - o results in persistent or significant disability or incapacity;
  - o consists of a congenital anomaly or birth defect.
- **Important Medical Events (IME) & Pregnancy:** Events that may not be immediately

life-threatening or result in death or hospitalisation but may jeopardise the participant or may require intervention to prevent one of the other outcomes listed in the definition above should also be considered serious. Although not a serious adverse event, any unplanned pregnancy will also be reported via the SAE reporting system.

10.2.1. Reporting Responsibilities

KCL and SLaM have delegated the delivery of the Sponsor's responsibility for Pharmacovigilance (as defined in Regulation 5 of the Medicines for Human Use (Clinical Trials) Regulations 2004 to the King's Health Partners Clinical Trials Office (KHP-CTO).

All SAEs, SARs and SUSARs will be reported immediately by the Chief Investigator (and certainly no later than 24hrs) to the KHP-CTO and Sponsor R&D office in accordance with the current Pharmacovigilance Policy.

The KHP-CTO will report SUSARs to the relevant ethics committee and the regulatory authority MHRA.

Reporting timelines are as follows:

- SUSARs which are fatal or life-threatening must be reported not later than 7 days after the sponsor is first aware of the reaction. Any additional relevant information must be reported within a further 8 days;
- SUSARs that are not fatal or life-threatening must be reported within 15 days of the sponsor first becoming aware of the reaction.

The Chief Investigator and KHP-CTO (on behalf of the co-sponsors) will submit a Development Safety Update Report (DSUR) relating to this trial IMP, to the MHRA and REC annually.

10.3. Adverse events that do not require reporting

All adverse events will be reported from time of consent to final follow-up.

10.4. Premature Termination of the Trial

The trial may be prematurely discontinued by the Sponsor, Chief Investigator or Regulatory Authority on the basis of new safety information or for other reasons given by the trial's research team, regulatory authority or ethics committee concerned.

If the trial is prematurely discontinued, active participants will be informed, and no further participant data will be collected. The Competent Authority and REC will be informed within 15 days of the early termination of the trial. There are no pre-specified treatment-stopping rules.

Dosing Suspension Criteria

Dosing may be temporarily suspended (for the individual or all participants on decision by the Trial Steering Committee (TSC)) under any one of the following objective criteria: occurrence of any serious adverse event (SAE) related to the investigational medicinal product (IMP); observation of the same severe adverse reaction in two or more participants; or upon decision by the TSC or Chief Investigator based on safety concerns. Should dosing suspension occur, appropriate escalation procedures will be implemented, including implementation of Urgent

Safety Measures or major protocol amendments as deemed necessary by the regulatory and oversight framework.

11. Statistics

11.1. Sample Size

No formal sample size calculation is computed as this is a pharmacokinetic study. Treatment selection will be made based on complete data of 12 participants (with five responses from each participant, each for an arm) at stage 1. With an expected drop-out rate of 20%, we expect to randomise 15 participants. This sample size exceeds the EMA recommendations for minimum sample size in pharmacokinetic studies for comparing formulations, providing robust data for analysis.

Stage 2 will recruit 30-35 participants, depending on the dropout rate observed in stage 1. A cross-over design will be implemented in stage 2 where the number of arms depend on the findings from stage 1. Participants that partake in stage 1 will not be eligible to also participate in Stage 2.

11.1.1. Power analysis

We will report post-hoc power analysis when studying the differences in bioavailability between the buccal, IM, and IN formulations of naloxone.

11.2. Randomisation

This study will employ an open-label, within-subject design with repeated sessions. Each participant in stage 1 of the study will receive all five treatment conditions in a random sequence.

Treatment allocation ordering for participants will be in a randomised order, following the randomisation schedule and process as detailed in section 7.5.

As this is an open-label study, there is no blinding involved. Both the investigators and the participants will be aware of the treatment allocation for each session.

11.3. Analysis

At the end of stage 1, $AUC_{0\text{-}15}$ of all arms will be computed for making comparisons to decide if further evaluation of IMPs will continue in stage 2. See decision criteria in section 5.4.

All the following analyses will be conducted when the study either terminates at stage 1 or at stage 2. Data will be stratified by stage if the following analysis is conducted at the end of stage 2.

<u>Statistical analysis plan:</u>

- Pharmacokinetics parameters:
  Pharmacokinetic parameters (C_{max}, T_{max}, AUC, $t_{1/2,\ T50\%}$) will be summarized descriptively. Continuous outcomes will be reported as means with standard deviation. Categorical outcomes will be reported as frequencies. Individual participant's

pharmacokinetic data will be presented in tables and graphs. The plasma concentration-time data will be subject to non-compartmental pharmacokinetic analysis using appropriate computer software applications. Linear and log-linear plots will be presented. To assess bioavailability between the formulations, log-transformed AUC and C_{max} observations will be entered into a linear mixed model to account for the repeated measures and between-subject conditions. Linear contrasts representing the difference between conditions will be expressed as a ratio of geometric means along with 95% CI and inference based on $p < 0.05$.

Protocol violations:

- All protocol violations, including inclusion/exclusion criteria violations and violations during the trial, will be listed, even if they are believed to influence any of the results.

Demographics:

- Demographic and baseline characteristics (including sex, age, BMI, and other relevant parameters) will be summarized using descriptive statistics for the overall study population.

Safety measures:

- Summary tables showing vital signs e.g. body temperature, respiratory rate, pulse rate, and systolic and diastolic blood pressure will be provided at each time point and changes from baseline.
- Individual physical examinations will be listed and summarised at each study time point.
- Important Medical Events, drugs and pregnancy test results during the study will be listed.

Disintegration time

- In vivo disintegration times of the buccal naloxone wafers will be summarized descriptively compared to in vitro data.

Laboratory results:

- Laboratory parameters including haematology, blood chemistry, and urinalysis will be listed accompanied by an indication if the parameter is outside the reference range. A summary of all data outside the reference range of the clinical laboratory will be provided. Clinical laboratory data will be presented descriptively, where applicable.

Adverse events:

- A listing of all individual AEs, AR, UAR, SAE, SAR and SUSAR will be provided. Summary tables of the TEAEs will be presented by the system organ class based on the MedDRA terminology list (preferred terms): 1 containing the number of TEAEs (frequency of occurrence, number of participants experiencing the event) by treatment and 1 containing the number of drug related TEAEs (frequency of occurrence, number of participants experiencing the event) per treatment. Additional tables of total counts by treatment and relationship and by treatment and severity will be given.

Missing data handling:

- This study will employ a complete case analysis approach, where only data from participants with full observations for a given endpoint will be included in the primary analysis for that endpoint. However, in the event of early withdrawal, any data already obtained (such as pharmacokinetic and safety data from completed visits) will be retained by the study and may be used in secondary analyses and presented in

publications, where scientifically appropriate. For participants who withdraw after completing at least one but not all experimental visits, their data from completed visits may be included in per-visit analyses.

12. Trial Management Group (TMG)

The TMG will include the Chief Investigator, co-investigators, the statistician, and the trial manager. The TMG will be responsible for overseeing the trial. Since it's a small pharmacokinetic study which is expected to be completed within a few months, we will meet on an ad hoc basis as and when any issues with the trial arise.

The TMG will review recruitment figures, SAEs and substantial amendments to the protocol prior to submission to the REC and/or MHRA.

13. Trial Steering Committee

The Trial Steering Committee (TSC) will provide overall supervision of the trial, ensuring it is conducted according to the protocol and in compliance with Good Clinical Practice (GCP) requirements. The TSC will monitor progress of the trial and recommend any appropriate amendments/actions for the trial as necessary.

For this small Phase 1 pharmacokinetic study with a well-characterized medication, a separate Data Monitoring Committee will not be established. All data monitoring responsibilities will be conducted by the Trial Management Group with oversight from the TSC. The TSC will review safety data after stage 1 before making decisions about progression to stage 2, ensuring appropriate safety monitoring throughout the trial.

14. Direct Access to Source Data and Documents

The Investigator(s) will permit trial-related monitoring, audits, REC review, and regulatory inspections by providing the Sponsors, Regulators and REC direct access to source data and other documents, such as participant consent documents, medical histories, laboratory results, relevant correspondence, and participant questionnaires.

15. Ethics & Regulatory Approvals

The trial will be conducted in compliance with the principles of the Declaration of Helsinki (1996), the principles of GCP and in accordance with all applicable regulatory requirements including but not limited to the Research Governance Framework and the Medicines for Human Use (Clinical Trial) Regulations 2004, as amended in 2006 and any subsequent amendments.

This protocol and related documents will be submitted for review to Health Research Authority (HRA), REC, and to the Medicines and Healthcare products Regulatory Agency (MHRA) for Clinical Trial Authorisation. Any subsequent protocol amendments will be submitted to the REC, HRA and Regulatory Authorities for approval as appropriate.

Prior to site recruitment the Chief Investigator/Principal Investigator or designee must receive local site approvals e.g. NHS permission in writing from the Trust Research & Development (R&D) known as confirmation of capability and capacity.

The Chief Investigator will submit a final report at conclusion of the trial to the KHP-CTO (on behalf of the Sponsor) and the REC within the timelines defined in the Regulations. The KHP-CTO or delegate will upload the final report to a publicly registered database on behalf of the Sponsor.

16. Quality Assurance

This is a small pharmacokinetic study with a short duration and short follow-up where the majority of outcome data will not be available until after laboratory analysis. The benefits of data audit are therefore limited. Data will be made available for inspection and monitoring by relevant authorities on request.

Monitoring of this trial will be to ensure compliance with Good Clinical Practice and scientific integrity will be managed and oversight retained, by the KHP-CTO Quality Team.

17. Data Management

The study will adhere to KCL's policy on data management, security and sharing. Participants will provide mobile phone numbers so that we can contact them during the study. If a participant requests, they will be contacted with the study outcome.

The Chief Investigator will act as custodian for the study data and all participant data will be pseudo-anonymised.

- All pseudo-anonymised data will be stored on a password-protected computer.
- Data will be collected using source data worksheets (paper questionnaires).
- Each study participant will be given a study ID.
- Source documents will be named with this ID.
- The source documents will be kept in locked cabinets. Study data will be transcribed to the Elsevier MACRO EDC system.
- All data will have restricted access with full audit trails for all activity

All shared research data will undergo pseudo-anonymization following ICH-GCP guidelines, with personal identifiers replaced by unique study ID. The primary research data available for sharing will include pseudo-anonymized pharmacokinetic data, non-identifiable demographic information, clinical observations, safety and tolerability measurements, and laboratory results, all documented in a standardized format suitable for future analysis Any personal data collected, will be stored and accessible for over 3 years after the study has ended. Research data generated by the study will be stored for 25 years.

All trial data will be stored in line with the Medicines for Human Use (Clinical Trials) Amended Regulations 2006 and the Data Protection Act and archived in line with the Medicines for Human Use (Clinical Trials) Amended Regulations 2006 as defined in the KHP-CTO Archiving Standard Operating Procedure (SOP). Regular backups of data will be made, retaining more than one copy of data.

All essential documents relating to the clinical study must be archived in accordance with the KHP-CTO SOP and the requirements of the UK Regulations as follows:

- Site Documentation: For at least 25 years after the completion of a clinical study, as defined by the Regulations.
- Trial Master File and all Essential Documentation: For a minimum of 25 years or until at least 2 years after the last approval of a marketing application in a region where the ICH guideline applies.

Data resulting from this study will be reported and disseminated at international conferences, in peer-reviewed scientific journals, publications on website and submission to regulatory authorities. Where appropriate, the results will be disseminated to the general public by means of press releases, posts on social media and at public engagement events; in all cases, data will be non-identifiable and individual participants will not be identifiable in publications.

17.1. Access to the final trial dataset

The final dataset of the trial will be made available to the study investigators and the trial statistician.

17.2. Data management in the event of early withdrawal

In the event of early withdrawal of a participant, any data already obtained, such as pharmacokinetic and safety, can be retained by the study and may be used in analyses and presented in publications.

18. Publication Policy

Data arising from the trial will be owned by KCL. It is intended that the results of the study will be reported and disseminated at international conferences and in peer-reviewed scientific journals. Where appropriate, the results will be disseminated to the general public by means of press releases, posts on social media and at public engagement events. The Consort Guidelines and checklist will be reviewed prior to generating any publications for the trial. Individual participants will not be identifiable in publications.

19. Insurance / Indemnity

Co-Sponsors KCL and SLaM insurance and indemnity schemes apply.

20. Financial Aspects

Funding to conduct the trial is provided by a grant awarded following competitive tendering and independent peer review process by The Scottish Chief Scientist’s Office through the SHIP (Scottish Health and Industry Partnership) initiative to support innovative strategies to address the challenge of drug-related deaths. In addition, supplementary funding has been provided by the pharmaceutical company Accord Healthcare.

The study drug and formulation will be provided by the manufacturer Catalent, from their Swindon UK facility, and using their established Zydis® technology platform, for which funding has been provided by the Scottish Chief Scientist's Office.

20.1. Payment

Participants will be reimbursed for their time at a rate of £16.00 per hour, reflecting the total time commitment of 25 hours for the five experimental visits in stage 1, totalling £400, in addition to £20 for completion of the screening visit. Upon successful completion of stage 1 components, participants will receive an additional completion bonus payment of £100, resulting in a total compensation payment of £520.00 for each volunteer. This payment of £520.00 for successful completion of stage 1 components is inclusive of all travel expenses to and from the clinical trial facility (no additional travel reimbursement will be provided).

For Stage 2 participants, the payment will be proportional to the number of experimental visits required, which will be determined after analysis of Stage 1 results. Punctual attendance for all blood tests is crucial to the study, and payment is contingent on completing all required aspects.

Participants who complete the screening visit but are unable to begin experimental visits of stage 1 will receive £20 as partial compensation. In case of withdrawal, participants will be reimbursed for the proportion of the study they have completed using a pro-rata payment.

21. Archiving

At the end of this trial, all trial data will be stored in line with the Medicines for Human Use (Clinical Trials) Amended Regulations 2006 and the 2018 Data Protection Act and archived in line with the Medicines for Human Use (Clinical Trials) Amended Regulations 2006 as defined in the Co-Sponsors Archiving Standard Operating Procedure (SOP).

22. Signatures

PROFESSOR SIR JOHN STRANG	John Strang	24st OCT 2025
Chief Investigator	Signature	Date
DR KIM LEE		24st OCT 2025
Statistician	Signature	Date

REFERENCES

1. WHO Opioid Overdose: Reducing Preventing and Opioid Overdose Mortality. *United Nations* 2013, 1–28.
2. Centers for Disease Control and Prevention (CDC) Opioid Overdose: Opioid Basics Available online: https://www.who.int/news-room/fact-sheets/detail/opioid-overdose.
3. Dhaliwal, A.; Gupta, M. Physiology , Opioid Receptor Available online: https://www.ncbi.nlm.nih.gov/books/NBK546642/.
4. Saari, T.I.; Strang, J.; Dale, O. Clinical Pharmacokinetics and Pharmacodynamics of Naloxone. *Clin Pharmacokinet* 2024, *63*, 397–422, doi:10.1007/s40262-024-01355-6.
5. E., G. The 1982 John Scott Award Goes to Jack Fishman and Harold Blumberg for Synthesis and Investigations on Naloxone. *Essay Inf. Sci* 1983, 121–130.
6. Strang, J.; McDonald, R.; Alqurshi, A.; Royall, P.; Taylor, D.; Forbes, B. Naloxone without the Needle - Systematic Review of Candidate Routes for Non-Injectable Naloxone for Opioid Overdose Reversal. *Drug Alcohol Depend* 2016, *163*, 16–23, doi:10.1016/j.drugalcdep.2016.02.042.
7. Lynn, R.R.; Galinkin, J. Naloxone Dosage for Opioid Reversal: Current Evidence and Clinical Implications. *Ther Adv Vaccines* 2018, *9*, 63–88, doi://doi.org/10.1177/2042098617744161 DOI: 10.1177/ https://doi.org/10.1177/20420986.
8. Strang, J.; McDonald, R.; Campbell, G.; Degenhardt, L.; Nielsen, S.; Ritter, A.; Dale, O. Take-Home Naloxone for the Emergency Interim Management of Opioid Overdose: The Public Health Application of an Emergency Medicine. *Drugs* 2019, *79*, 1395–1418, doi:10.1007/s40265-019-01154-5.
9. Elzey, M.J.; Fudin, J.; Edwards, E.S. Take-Home Naloxone Treatment for Opioid Emergencies: A Comparison of Routes of Administration and Associated Delivery Systems. *Expert Opin Drug Deliv* 2017, *14*, 1045–1058, doi:10.1080/17425247.2017.1230097.
10. Wermeling, D.P. Review of Naloxone Safety for Opioid Overdose: Practical Considerations for New Technology and Expanded Public Access. *Ther Adv Drug Saf* 2015, *6*, 20–31, doi:10.1177/2042098614564776.
11. WHO Expert Committee The Use of Essential Drugs. Technical Report Series 685. 1983.
12. McAuley, A.; Aucott, L.; Matheson, C. Exploring the Life-Saving Potential of Naloxone: A Systematic Review and Descriptive Meta-Analysis of Take Home Naloxone (THN) Programmes for Opioid Users. *International Journal of Drug Policy* 2015, *26*, 1183–1188, doi:10.1016/j.drugpo.2015.09.011.
13. Bell, J.; Strang, J. Medication Treatment of Opioid Use Disorder. *Biol Psychiatry* 2020, *87*, 82–88, doi:10.1016/j.biopsych.2019.06.020.
14. Strang, J.; McDonald, R. *Preventing Opioid Overdose Deaths with Take-Home Naloxone*; 2016;
15. Bennett, A.S.; Elliott, L. Naloxone's Role in the National Opioid Crisis—Past Struggles, Current Efforts, and Future Opportunities. *Translational Research* 2021, *234*, 43–57, doi:10.1016/j.trsl.2021.03.001.
16. Volkow, N.D.; Frieden, T.R.; Hyde, P.S.; Cha, S.S. Medication-Assisted Therapies — Tackling the Opioid- Overdose Epidemic Nora. *New England Journal of Medicine* 2014, *370*, 2063–2066, doi:10.1056/NEJMp1402780.
17. Doe-Simkins, M.; Walley, A.Y.; Epstein, A.; Moyer, P. Saved by the Nose: Bystander-Administered Intranasal Naloxone Hydrochloride for Opioid Overdose. *Am J Public Health* 2009, *99*, 788–791, doi:10.2105/AJPH.2008.146647.
18. Ethypharm Naloxone Hydrochloride Injections: Opioid Overdose Available online: http://www.prenoxadinjection.com/hcp/abt_naloxone.html.
19. Parsons, G. Nyxoid (Naloxone) Nasal Spray for Opioid Overdose. *Prescriber* 2019, *30*, 38–39, doi:10.1002/psb.1764.
20. McDonald, R.; Campbell, N.D.; Strang, J. Twenty Years of Take-Home Naloxone for the Prevention of Overdose Deaths from Heroin and Other Opioids—Conception and Maturation. *Drug Alcohol Depend* 2017, *178*, 176–187, doi:10.1016/j.drugalcdep.2017.05.001.
21. Evoy, K.E.; Hill, L.G.; Davis, C.S. Considering the Potential Benefits of Over-the-Counter Naloxone. *Integr Pharm Res Pract* 2021, *Volume 10*, 13–21, doi:10.2147/iprp.s244709.
22. Miller, N.M.; Campbell, C.; Shorter, G.W. Barriers and Facilitators of Naloxone and Safe Injection Facility Interventions to Reduce Opioid Drug-Related Deaths: A Qualitative Analysis. *International Journal of Drug Policy* 2023, *117*, 104049, doi:10.1016/j.drugpo.2023.104049.
23. Kozak, Z.; Ciccarone, D.; Thrul, J.; Cole, T.O.; Pappas, A.L.; Greenblatt, A.D.; Welsh, C.; Yoon, M.; Gann, D.; Artigiani, E.E.; et al. Harm Reduction Behaviors Are Associated with

Carrying Naloxone among Patients on Methadone Treatment. *Harm Reduct J* 2023, *20*, 1–6, doi:10.1186/s12954-023-00745-6.
24. Burton, G.; McAuley, A.; Schofield, J.; Yeung, A.; Matheson, C.; Parkes, T. A Systematic Review and Meta-Analysis of the Prevalence of Take-Home Naloxone (THN) Ownership and Carriage. *International Journal of Drug Policy* 2021, *96*, 103298, doi:10.1016/j.drugpo.2021.103298.
25. McDonald, R.; Lorch, U.; Woodward, J.; Bosse, B.; Dooner, H.; Mundin, G.; Smith, K.; Strang, J. Pharmacokinetics of Concentrated Naloxone Nasal Spray for Opioid Overdose Reversal: Phase I Healthy Volunteer Study. *Addiction* 2018, *113*, 484–493, doi:10.1111/add.14033.
26. Mundin, G.; McDonald, R.; Smith, K.; Harris, S.; Strang, J. Pharmacokinetics of Concentrated Naloxone Nasal Spray over First 30 Minutes Post-Dosing: Analysis of Suitability for Opioid Overdose Reversal. *Addiction* 2017, *112*, 1647–1652, doi:10.1111/add.13849.
27. Pather, S.I.; Rathbone, M.J.; Şenel, S. Current Status and the Future of Buccal Drug Delivery Systems. *Expert Opin Drug Deliv* 2008, *5*, 531–542, doi:10.1517/17425247.5.5.531.
28. Sattar, M.; Sayed, O.M.; Lane, M.E. Oral Transmucosal Drug Delivery - Current Status and Future Prospects. *Int J Pharm* 2014, *471*, 498–506, doi:10.1016/j.ijpharm.2014.05.043.
29. Hussain, M.A.; Aungst, B.J.; Kearney, A.; Shefter, E. Buccal and Oral Bioavailability of Naloxone and Naltrexone in Rats. *Int J Pharm* 1987, *36*, 127–130, doi:10.1016/0378-5173(87)90147-5.
30. Johnson, R.; Azimi, N.; Schnipper, E.F. PHARMACOKINETICS OF TRANSBUCCAL SWAB-ADMINISTERED NALOXONE-HCL USING A NOVEL PLANT-. *biorxiv* 2021, 1–20.
31. Cohen, M. Behavioural Effects after High Dose Naloxone Administration to Normal Volunteers. *Lancet* 1981, *2*.
32. Volavka, J.; James, B.; Reker, D.; Pollock, V.; Cho, D. Electroencephalographic and Other Effects of Naloxone in Normal Men. *Life Sci* 1979, *25*, 1267–1272, doi:https://doi.org/10.1016/0024-3205(79)90471-5.
33. Alqurshi, A.; Kumar, Z.; McDonald, R.; Strang, J.; Buanz, A.; Ahmed, S.; Allen, E.; Cameron, P.; Rickard, J.A.; Sandhu, V.; et al. Amorphous Formulation and in Vitro Performance Testing of Instantly Disintegrating Buccal Tablets for the Emergency Delivery of Naloxone. *Mol Pharm* 2016, *13*, 1688–1698, doi:10.1021/acs.molpharmaceut.6b00096.
34. McIntyre, J.; Robertson, S.; Norris, E.; Appleton, R.; Whitehouse, W.P.; Phillips, B.; Martland, T.; Berry, K.; Collier, J.; Smith, S.; et al. Safety and Efficacy of Buccal Midazolam versus Rectal Diazepam for Emergency Treatment of Seizures in Children: A Randomised Controlled Trial. *Lancet* 2005, *366*, 205–210, doi:10.1016/S0140-6736(05)66909-7.
35. Sudhakar, Y.; Kuotsu, K.; Bandyopadhyay, A.K. Buccal Bioadhesive Drug Delivery - A Promising Option for Orally Less Efficient Drugs. *Journal of Controlled Release* 2006, *114*, 15–40, doi:10.1016/j.jconrel.2006.04.012.
36. Balster, R.; Broers, B.; Buxton, J.; Dietze, P.; Horsburgh, K.; Jain, R.; Khan, N.; Kloek, W.; Razzahi, E.; Sawe, H.; et al. *Substance Use - Community Management of Opioid Overdose*; 2018;
37. McDonald, R.; Lorch, U.; Woodward, J.; Bosse, B.; Dooner, H.; Mundin, G.; Smith, K.; Strang, J. Pharmacokinetics of Concentrated Naloxone Nasal Spray for Opioid Overdose Reversal: Phase I Healthy Volunteer Study. *Addiction* 2018, *113*, 484–493, doi:10.1111/add.14033.
38. van Dorp, E. LA; Yassen, A.; Dahan, A. Naloxone Treatment in Opioid Addiction: The Risks and Benefits. *Expert Opin Drug Saf* 2007, *6*, 125–132, doi:10.1517/14740338.6.2.125.
39. (EPAR), E. public assessment report Nyxoid EPAR Summary. *European Medicines Agency Science Medicines Health* 2017, *44*.
40. Skulberg, A.K.; Tylleskar, I.; Nilsen, T.; Skarra, S.; Salvesen, Ø.; Sand, T.; Loftsson, T.; Dale, O. Pharmacokinetics and -Dynamics of Intramuscular and Intranasal Naloxone: An Explorative Study in Healthy Volunteers. *Eur J Clin Pharmacol* 2018, *74*, 873–883, doi:10.1007/s00228-018-2443-3.
41. Hikma Specialty USA Inc. Kloxxado (Naloxone HCl). *Full Prescribing Information. Columbus, OH: Hikma Specialty USA Inc* 2021.
42. Cohen, M.R.; Cohen, R.M.; Pickar, D.; Murphy, D.L.; Bunney, W.E. Physiological Effects of High Dose Naloxone Administration to Normal Adults. *Life Sci* 1982, *30*, 2025–2031, doi:10.1016/0024-3205(82)90443-X.
